# The neurophysiological effect of mild hypothermia in gyrencephalic brains submitted to ischemic stroke and spreading depolarizations

**DOI:** 10.3389/fnins.2024.1302767

**Published:** 2024-03-14

**Authors:** Roberto Díaz-Peregrino, Modar Kentar, Carlos Trenado, Renán Sánchez-Porras, Pablo Albiña-Palmarola, Francisco L. Ramírez-Cuapio, Daniel San-Juan, Andreas Unterberg, Johannes Woitzik, Edgar Santos

**Affiliations:** ^1^Department of Neurosurgery, University Hospital Heidelberg, Ruprecht-Karls-University Heidelberg, Heidelberg, Germany; ^2^Departement of Neurosurgery, Städtisches Klinikum Braunschweig gGmbH, Braunschweig, Germany; ^3^Heinrich Heine University, Medical Faculty, Institute of Clinical Neuroscience and Medical Psychology, Düsseldorf, Germany; ^4^Institute for the Future of Education Europe, Tecnológico de Monterrey, Cantabria, Spain; ^5^Department of Neurosurgery, Evangelisches Krankenhaus, Carl von Ossietzky University Oldenburg, Oldenburg, Germany; ^6^Neuroradiologische Klinik, Klinikum Stuttgart, Stuttgart, Germany; ^7^Medizinische Fakultät, Universität Duisburg-Essen, Essen, Germany; ^8^Department of Anatomy, School of Medicine, Pontificia Universidad Católica de Chile, Santiago, Chile; ^9^Epilepsy Clinic, National Institute of Neurology and Neurosurgery, Manuel Velasco Suárez, Mexico City, Mexico

**Keywords:** spreading depolarization, stroke progression, ECoG recording, mild hypothermia, power spectrum of frequency bands

## Abstract

**Objective:**

Characterize the neurophysiological effects of mild hypothermia on stroke and spreading depolarizations (SDs) in gyrencephalic brains.

**Methods:**

Left middle cerebral arteries (MCAs) of six hypothermic and six normothermic pigs were permanently occluded (MCAo). Hypothermia began 1 h after MCAo and continued throughout the experiment. ECoG signals from both frontoparietal cortices were recorded. Five-minute ECoG epochs were collected 5 min before, at 5 min, 4, 8, 12, and 16 h after MCAo, and before, during, and after SDs. Power spectra were decomposed into fast (alpha, beta, and gamma) and slow (delta and theta) frequency bands.

**Results:**

In the vascular insulted hemisphere under normothermia, electrodes near the ischemic core exhibited power decay across all frequency bands at 5 min and the 4th hour after MCAo. The same pattern was registered in the two furthest electrodes at the 12th and 16th hour. When mild hypothermia was applied in the vascular insulted hemispheres, the power decay was generalized and seen even in electrodes with uncompromised blood flow. During SD analysis, hypothermia maintained increased delta and beta power during the three phases of SDs in the furthest electrode from the ischemic core, followed by the second furthest and third electrode in the beta band during preSD and postSD segments. However, in hypothermic conditions, the third electrode showed lower delta, theta, and alpha power.

**Conclusion:**

Mild hypothermia attenuates all frequency bands in the vascularly compromised hemisphere, irrespective of the cortical location. During SD formation, it preserves power spectra more significantly in electrodes further from the ischemic core.

## 1 Introduction

**Figure 1:**
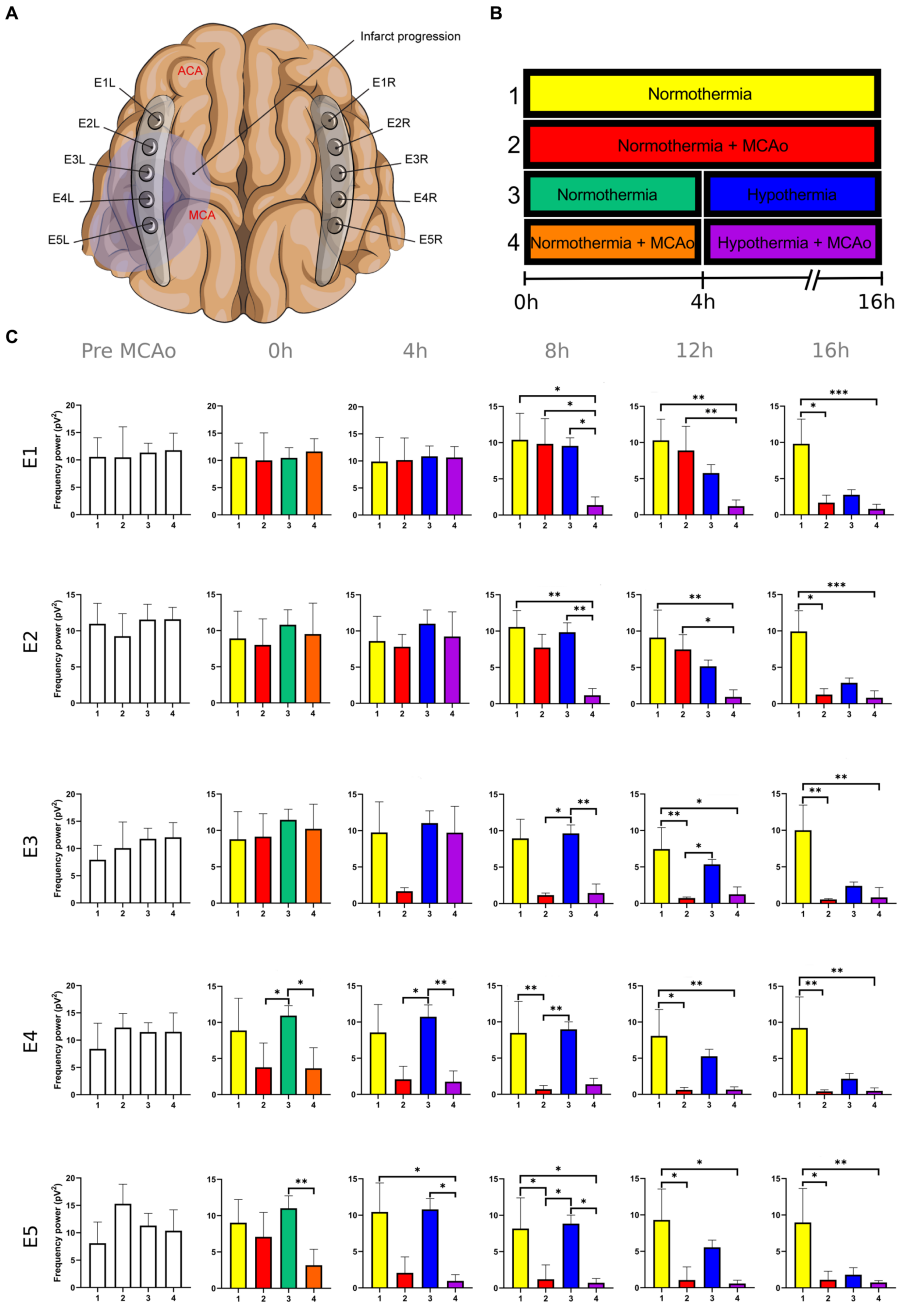
Power spectra of the delta band in the insulted and healthy hemispheres under normothermic and hypothermic conditions. **(A)** Ten electrodes were utilized for the recording of ECoG signals, with five electrodes (E1R-E5R) placed on the unaffected right hemisphere and another set of five electrodes (E1L-E5L) positioned on the insulted left hemisphere. It is speculated that E1 was placed over the ACA region, while E5 was placed over the cerebral cortex close to the MCAo. **(B)** The statistical analysis included the comparison of four scenarios: Two conditions within the right hemisphere, unaffected by MCAo, denoted as the healthy hemisphere under normothermia (1) and the healthy hemisphere under hypothermia (3), and two more conditions in the left hemisphere with MCAo, identified as the insulted hemisphere under normothermia (2) and the insulted hemisphere under hypothermia (4). **(C)** The multiple comparison computed throughout the Kruskal-Wallis and Dunn’s tests within the 4 conditions were performed at time points 0 h, 4 h, 8 h, 12 h, and 16 h. Therefore a consecutive Holm-Bonferroni test was computed for the multiple comparison adjustment over time. The statistically significant changes in the power spectrum of the frequency bands within conditions are represented as * when the value of *p* was equal to ≤0.05, ** when it was equal to ≤0.01, and *** when it was equal to ≤0.001. pV^2^, squared picovolts.

Hypothermia has been suggested as a therapeutic approach to stroke based on previous ischemic animal model studies, which report a decline in motor sequelae, edema attenuation, and lowered inflammatory response (Colbourne et al., 2000; Kollmar et al., 2007; Clark et al., 2008). In addition, hypothermia favors infarct size reduction between 30 and 44% in rats and pigs (van der Worp et al., 2007; Zhang et al., 2007). We have corroborated these findings, reporting the shrinkage of the infarct volume in pigs submitted to cold temperatures (6.0 ± 1.0 cm^3^) compared to normothermic animals (9 ± 0.8 cm^3^) (Figure 1; Kentar et al., 2023).

In addition to the study of stroke, it is vital to understand the aggravating factors that facilitate the growth of the necrotic core and penumbra zones, such as in spreading depolarizations (SDs) (Dreier et al., 2009; Ayata and Lauritzen, 2015). Among the physiological factors, the incidence of SDs and temperature are interrelated. There is an increase of 0.2°C 25 min before the onset of SD and 0.4°C when a cluster occurs. Furthermore, a higher probability of SDs occurrence has been reported during episodes of brain temperature over 38°C (Schiefecker et al., 2018). Hypothermia prevents damage in the normally perfused cortex by delaying the appearance of SD (Sasaki et al., 2009). According to our observations, mean SD frequency (1.0 ± 0.7 vs. 3.5 ± 2.1 per hour) and mean SD expansion (41.9 ± 21.8% vs. 73.2 ± 5.2%) were diminished in hypothermic than normothermic brains (Kentar et al., 2023).

The outcomes of clinical trials, however, are mixed, with the variability in results mainly explained by differences in the insult severity, time, and doses of hypothermia administration (Clifton et al., 2011; van der Worp et al., 2014; Kuczynski et al., 2020). Current literature suggests appropriate doses and time windows can be tailored depending on biomarkers (Lyden, 2020). Based on our previous report (Kentar et al., 2022), we expect that power spectrum changes at different frequency bands may be helpful as a marker of low brain activity coupled with reduced brain temperature. The frequency bands as biomarkers can lead to the efficient application of mild hypothermia and the avoidance of the adverse effects of extremely low temperatures.

However, an accurate analysis of the frequency band alterations induced by hypothermia that lead to infarct evolution remains ongoing. Previous EEG physiological monitoring studies have reported theta and beta band changes at 33.5°C, resembling a non-REM sleep rhythm, with a progressive slowing down of the frequency band and signal amplitude, leading eventually to an isoelectric signal under more profound hypothermia at 25°C (Pearcy and Virtue, 1960; Deboer and Tobler, 1995; Kochs, 1995; Abend et al., 2009; Mahfooz et al., 2017; Myers et al., 2017).

In this regard, our first efforts were focused on investigating the natural history of ischemic stroke from the neurophysiological aspect. We proposed a core-penumbra map, where the electrodes in the core close to the middle cerebral artery occlusion (MCAo) lost all frequency bands immediately after the MCAo. Meanwhile, the electrodes in the penumbra exhibited an immediate decay in the fast frequencies accompanied by the gradual ceasing of the slower bands. On the contrary, the electrodes placed within the healthy cortex in the anterior cerebral artery (ACA) territory suffered a late decay in all the frequency bands. We also characterized the neurophysiological traits of the SDs according to the cortical location in the vicinity of MCAo. SDs provoked the sudden power slump of all the frequency bands in places where they were passing through, but a power recovery of the 5 frequencies was observed when the SDs left the healthy cortex supplied by the ACA (Kentar et al., 2022).

To understand the neurophysiological mechanism associated with mild hypothermia in stroke, we conducted a *post hoc* analysis of previously reported MCAo experiments in swine (Kentar et al., 2023). We used the ECoG recording signals to monitor the power changes of the frequency bands during the infarct evolution and the development of SDs. We hypothesized that (I) the complete establishment of mild hypothermia around 32°C at the 4th hour is accompanied by the silence of all the frequency bands regardless of the cortical location. Furthermore, we expect that (II) mild hypothermia counteracts the harmful effects of SD, impeding the collapse of some frequency bands during the development and expansion of SD over the cortex and promoting their power recovery after the migration of SD.

## 2 Materials and methods

The animal preparation, surgical procedure, temperature measurement, and infarct volume determination are extensively explained in our article (Kentar et al., 2023).

### 2.1 Experimental set-up

ECoG was recorded with a sampling rate of 200 Hz by utilizing a Powerlab 16/SP analog-to-digital converter (ADInstruments, Sydney, Australia). Two subdural five-contact platinum wall strip electrodes (Ad-tech, Racine, Wisconsin, USA) were employed. The zygomatic bone was used as a ground electrode. The filter on the alternating current (AC) recorder was set at 0.1 Hz. A notch filter (50 Hz) was applied to eliminate line disturbances. ECoG analysis and registration were carried out with LabChart v7 (ADInstruments).

### 2.2 ECoG recording

The ECoG recordings were scheduled to last 24 h, 1 h before MCAo and 23 h after MCAo. On the cortical surface, five-contact ECoG strips were positioned on either side, corresponding to the MCA and ACA areas, with 10 mm between each electrode. Ten electrodes were used to record ECoG signals: 5 electrodes (E1R-E5R) from the healthy right hemisphere and 5 electrodes (E1L-E5L) from the injured left hemisphere. Electrodes E5 were caudal and coincided with the MCA region, whereas electrodes E1 were rostral and corresponded to the ACA territory in the frontal hemisphere.

In the core-penumbra map proposed in our previous work (Kentar et al., 2022), we concur with the finding observed by Rabiller and collaborators (Rabiller et al., 2015), who registered the modification in cortical areas submitted to different levels of vascular depletion using EEG. Thus, it was possible to define three zones based on the electrographic patterns. The non-vascular compromised areas (CBF over 35 mL/100 g/min) display no modifications in the frequency bands. The penumbra (CBF between 35 to 12 mL/100 g/min) is defined by the early decay of fast frequencies (alpha, beta and gamma) and the late fall of the slow ones (delta and theta). The ischemic core (CBF lower than 10 mL/100 g/min) exhibits the suppression of all the frequency bands. The electrodes in all our experiments are precisely set according to the detection of the non-spreading depression observed in the caudal electrodes E5L and E4L (Dreier et al., 2017; Kentar et al., 2022), which also registered the early depression of all frequency bands as the ischemic core shows. Consequently, E3 and lately E2 recorded the electrographic patterns of penumbra, and E1 the patterns of a non-vascular compromised area.

We speculate that E1 registers the cortex supplied by ACA due to the mean infarct volume in the MCA territory of normothermic brains, which reached 9.00 ± 0.8 cm^3^ in our past work (Kentar et al., 2023). Based on the distance to the ischemic core studied in our last study of frequency band analysis (Kentar et al., 2022), E1 did not registered any modification in all the frequency bands right after the MCAo up to around the 4th and 8th hour of the ECoG recording, meaning that the vascular occlusion did not affect the brain cortex underneath E1 early, but the extension of the brain infarct reached vascular areas above the MCA in the late ECoG period, namely the ACA.

### 2.3 Frequency analysis

The infarct development was evaluated by collecting the 5-min ECoG signal epoch before MCAo and 5 min, 4, 8, 12, and 16 h after MCAo. SDs were registered in an AC recorder as a negative near-DC shift (NDCS) accompanied by a loss of power in the ECoG bands in nearby electrodes (Dreier et al., 2017). Before and following the NDCS of the SDs, 5-min signal segments were utilized for the SD assessment. Additionally, the NDCS were analyzed, lasting an average of 49.7 s (±12.3 s). For accurate SD assessment, the signal segments 5 min before and after the NDCS must be devoid of artifacts and other SDs. ECoG recording segments before, during, and following the NDCS were referred to as “preSD,” “SD,” and “postSD,” respectively (Kentar et al., 2022).

Power spectra of ECoG epochs corresponding to different experimental conditions were decomposed in various frequency bands by calculating the discrete 512-point Fourier transform (Hanning window) (Al-Fahoum and Al-Fraihat, 2014; Delimayanti et al., 2020; Kentar et al., 2022). The following power frequency bands were considered for each ECoG epoch: Slow frequencies: Delta (0.1–4 Hz) c and theta (4–7 Hz). Fast frequencies: Alpha (8–12 Hz), beta (13–31 Hz), and gamma (32–45 Hz). Customized MATLAB algorithms (MathWorks, Natick, MA) were used to compute the power spectrum of each frequency band (Kentar et al., 2022).

The power spectrum for each frequency band was computed every 4 h to analyze the neurophysiological dynamics during the progression of the infarct, encompassing four time points after the MCAo plus the baseline collected 5 min before the MCAo. Similarly, the power spectrum for each frequency band was calculated for each SD phase (preSD, SD, and post-SD).

### 2.4 Statistical analysis

**Figure 2:**
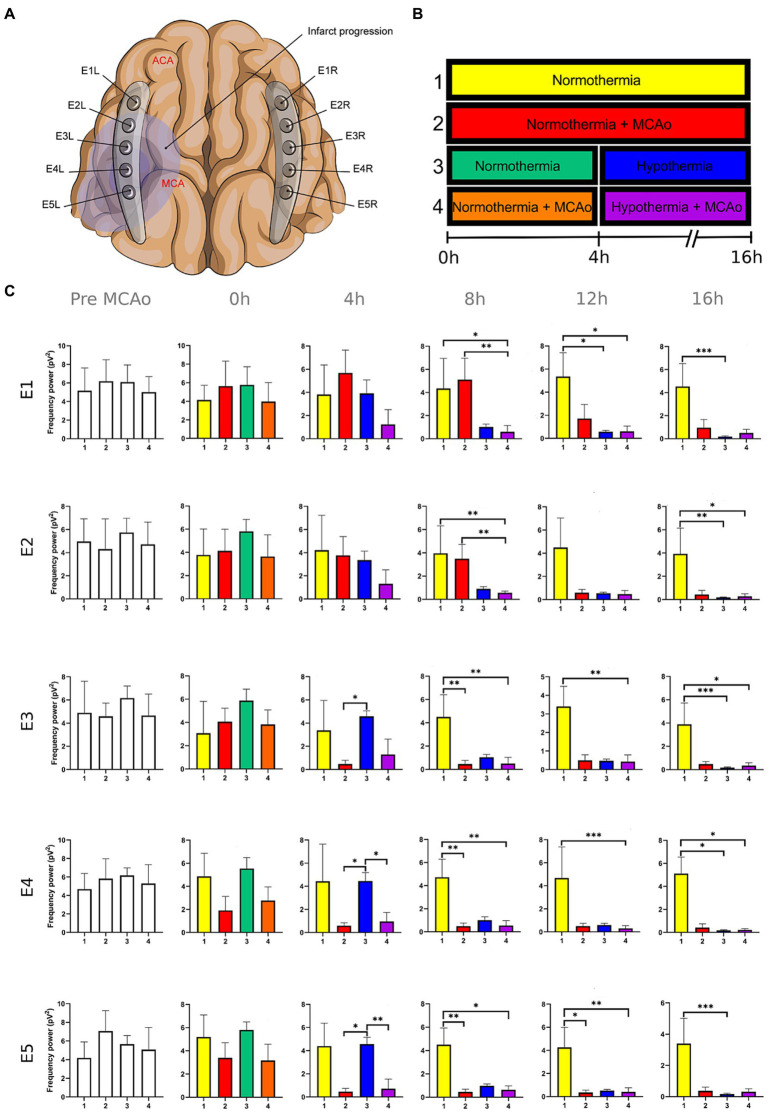
Power spectra of the theta band in the insulted and healthy hemispheres under normothermic and hypothermic conditions. **(A)** Ten electrodes were utilized for the recording of ECoG signals, with five electrodes (E1R-E5R) placed on the unaffected right hemisphere and another set of five electrodes (E1L-E5L) positioned on the insulted left hemisphere. It is speculated that E1 was placed over the ACA region, while E5 was placed over the cerebral cortex close to the MCAo. **(B)** The statistical analysis included the comparison of four scenarios: Two conditions within the right hemisphere, unaffected by MCAo, denoted as the healthy hemisphere under normothermia (1) and the healthy hemisphere under hypothermia (3), and two more conditions in the left hemisphere with MCAo, identified as the insulted hemisphere under normothermia (2) and the insulted hemisphere under hypothermia (4). **(C)** The multiple comparison computed throughout the Kruskal-Wallis and Dunn’s tests within the 4 conditions were performed at time points 0 h, 4 h, 8 h, 12 h, and 16 h. Therefore a consecutive Holm-Bonferroni test was computed for the multiple comparison adjustment over time. The statistically significant changes in the power spectrum of the frequency bands within conditions are represented as * when the value of p was equal to ≤0.05, ** when it was equal to ≤0.01, and *** when it was equal to ≤0.001. pV^2^, squared picovolts.

**Figure 3:**
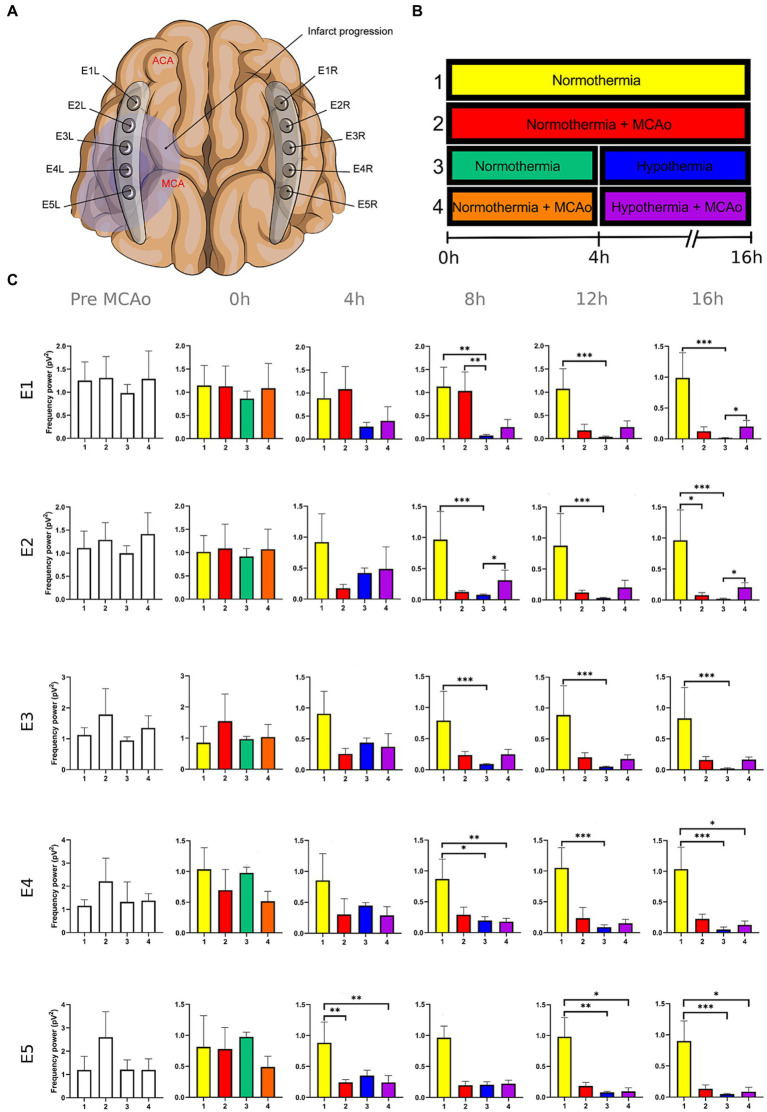
Power spectra of the alpha band in the insulted and healthy hemispheres under normothermic and hypothermic conditions. **(A)** Ten electrodes were utilized for the recording of ECoG signals, with five electrodes (E1R-E5R) placed on the unaffected right hemisphere and another set of five electrodes (E1L-E5L) positioned on the insulted left hemisphere. It is speculated that E1 was placed over the ACA region, while E5 was placed over the cerebral cortex close to the MCAo. **(B)** The statistical analysis included the comparison of four scenarios: Two conditions within the right hemisphere, unaffected by MCAo, denoted as the healthy hemisphere under normothermia (1) and the healthy hemisphere under hypothermia (3), and two more conditions in the left hemisphere with MCAo, identified as the insulted hemisphere under normothermia (2) and the insulted hemisphere under hypothermia (4). **(C)** The multiple comparison computed throughout the Kruskal-Wallis and Dunn’s tests within the 4 conditions were performed at time points 0 h, 4 h, 8 h, 12 h, and 16 h. Therefore a consecutive Holm-Bonferroni test was computed for the multiple comparison adjustment over time. The statistically significant changes in the power spectrum of the frequency bands within conditions are represented as * when the value of *p* was equal to ≤0.05, ** when it was equal to ≤0.01, and *** when it was equal to ≤0.001. pV^2^, squared picovolts.

**Figure 4:**
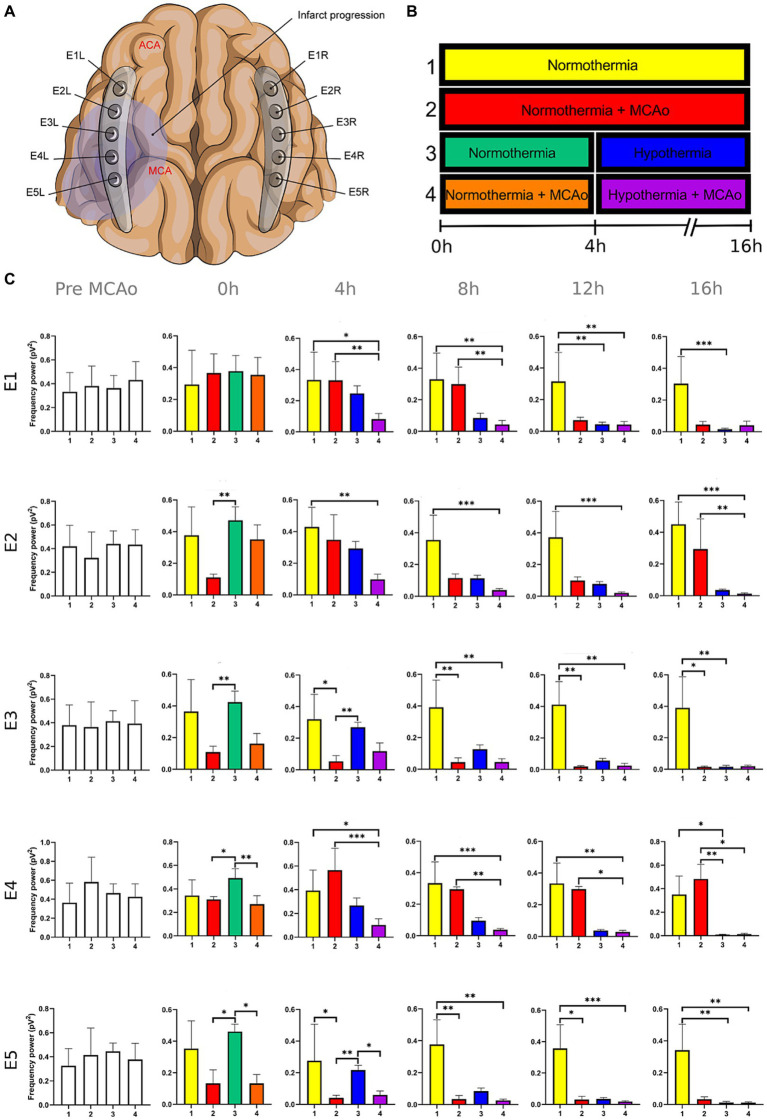
Power spectra of the beta band in the insulted and healthy hemispheres under normothermic and hypothermic conditions. **(A)** Ten electrodes were utilized for the recording of ECoG signals, with five electrodes (E1R-E5R) placed on the unaffected right hemisphere and another set of five electrodes (E1L-E5L) positioned on the insulted left hemisphere. It is speculated that E1 was placed over the ACA region, while E5 was placed over the cerebral cortex close to the MCAo. **(B)** The statistical analysis included the comparison of four scenarios: Two conditions within the right hemisphere, unaffected by MCAo, denoted as the healthy hemisphere under normothermia (1) and the healthy hemisphere under hypothermia (3), and two more conditions in the left hemisphere with MCAo, identified as the insulted hemisphere under normothermia (2) and the insulted hemisphere under hypothermia (4). **(C)** The multiple comparison computed throughout the Kruskal-Wallis and Dunn’s tests within the 4 conditions were performed at time points 0 h, 4 h, 8 h, 12 h, and 16 h. Therefore a consecutive Holm-Bonferroni test was computed for the multiple comparison adjustment over time. The statistically significant changes in the power spectrum of the frequency bands within conditions are represented as * when the value of *p* was equal to ≤0.05, ** when it was equal to ≤0.01, and *** when it was equal to ≤0.001. pV^2^, squared picovolts.

**Figure 5:**
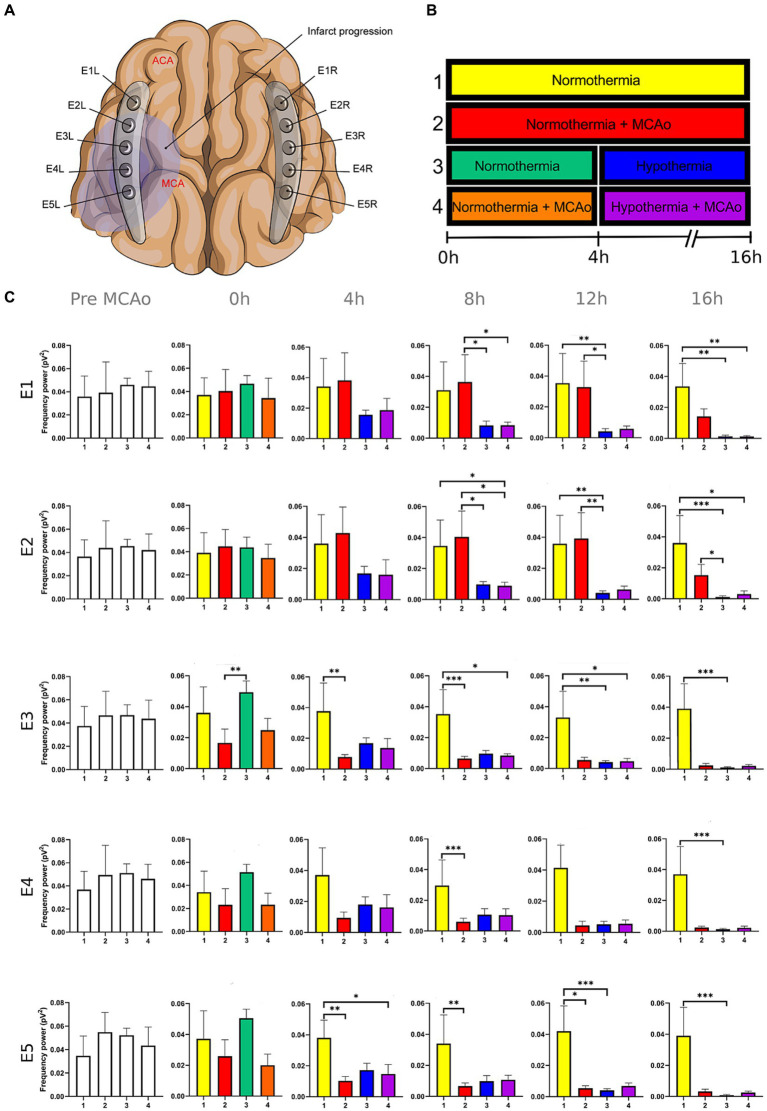
Power spectra of the gamma band in the insulted and healthy hemispheres under normothermic and hypothermic conditions. **(A)** Ten electrodes were utilized for the recording of ECoG signals, with five electrodes (E1R-E5R) placed on the unaffected right hemisphere and another set of five electrodes (E1L-E5L) positioned on the insulted left hemisphere. It is speculated that E1 was placed over the ACA region, while E5 was placed over the cerebral cortex close to the MCAo. **(B)** The statistical analysis included the comparison of four scenarios: Two conditions within the right hemisphere, unaffected by MCAo, denoted as the healthy hemisphere under normothermia (1) and the healthy hemisphere under hypothermia (3), and two more conditions in the left hemisphere with MCAo, identified as the insulted hemisphere under normothermia (2) and the insulted hemisphere under hypothermia (4). **(C)** The multiple comparison computed throughout the Kruskal-Wallis and Dunn’s tests within the 4 conditions were performed at time points 0 h, 4 h, 8 h, 12 h, and 16 h. Therefore a consecutive Holm-Bonferroni test was computed for the multiple comparison adjustment over time. The statistically significant changes in the power spectrum of the frequency bands within conditions are represented as * when the value of *p* was equal to ≤0.05, ** when it was equal to ≤0.01, and *** when it was equal to ≤0.001. pV^2^, squared picovolts.

The statistical analysis was performed using SPSS v25 (IBM, Armonk, NY), and the plots were made in GraphPad Prism 8.0.1 (GraphPad Software, San Diego, CA). Shapiro–Wilk analysis was used to determine the distribution of the data. The data displayed a non-normal distribution. As a result, the following non-parametric tests were carried out:

I. Differences in the power of frequency bands in the vascular insulted and healthy hemispheres under normothermic and hypothermic conditions for each timepoint: The power spectral analysis of the frequency bands was obtained at 0, 4th, 8th, 12th, and 16th hour after the MCAo, having as a baseline 5-min epoch before the MCAo. The study involved the comparison of four conditions. Specifically, two conditions were examined in the right hemisphere without MCAo: The healthy hemisphere under normothermia (1) and the healthy hemisphere under hypothermia (3). Additionally, two conditions from the left hemisphere with MCAo were analyzed: The insulted hemisphere under normothermia (2) and the insulted hemisphere under hypothermia (4). The Kruskal-Wallis test and Dunn’s test were conducted to identify variations in the power spectrum of the frequency bands among the 4 conditions for each timepoint precisely. After Dunn’s test, a Holm-Bonferroni adjustment was carried out to account for multiple comparisons across different time points (*p* = 0.05/6). As a result, the revised value of *p* was established at 0.0083. The outcomes obtained from Dunn’s test depicted in Figures 1–5; Supplementary Tables 1–5 are those with value of *p* less than 0.0083 in the overall iteration of the Kruskal-Wallis test.

II. Alterations in the power of the frequency bands after MCAo in the healthy and vascular insulted hemispheres under hypothermia over time: The power spectral analysis of the frequency bands was obtained at 0 h, 4 h, 8 h, 12 h, and 16 h after the MCAo, having as a baseline 5-min epoch before the MCAo. The Wilcoxon matched-pairs signed-rank test was used to compare the baseline with each timepoint, analyzing each condition separately (Supplementary Figures 1–4; Supplementary Tables 7–10).

**Figure 6:**
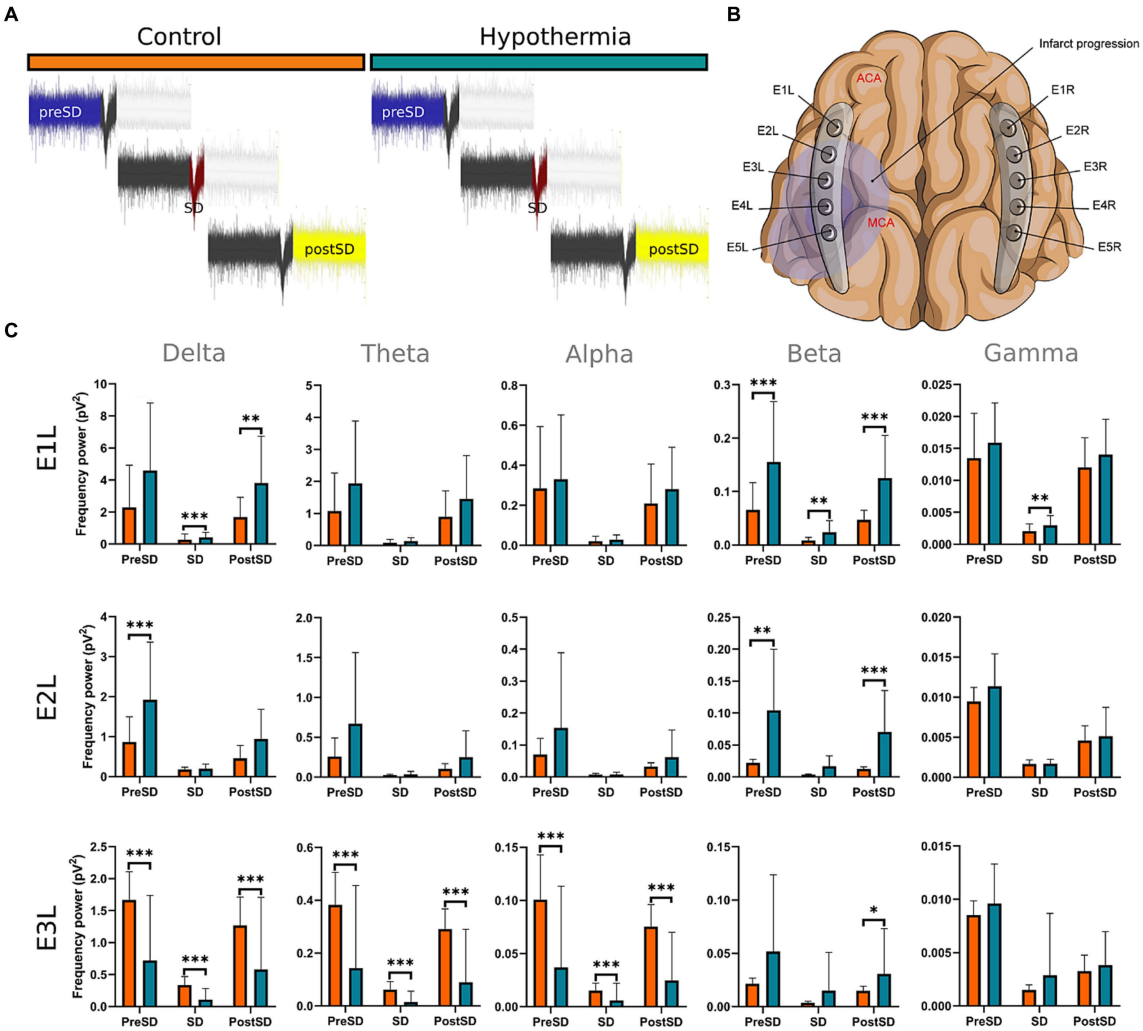
Power spectra of frequency bands before, during, and after the SD formation in the insulted hemisphere under normothermic and hypothermic conditions. **(A,B)** The SDs were obtained from E1 to E3. From each normothermic and hypothermic scenario, 27 from the E1,14 from E2, and 13 from E3 were analyzed. Each segment from the collected SD in the normothermia group was compared with its counterpart in the hypothermia arm **(C)**. The U-Mann Whitney test was performed for the comparison among the two conditions. A sequential Holm-Bonferroni test was performed to adjust for multiple comparisons over time. The statistically significant differences between conditions are represented as * when the value of *p* was equal to ≤0.05, ** when it was equal to ≤0.01, and *** when it was equal to ≤0.001. pV^2^, squared picovolts.

III. Changes in frequency bands during the development of SDs in the left insulted hemispheres under normothermic and hypothermic conditions: The Mann–Whitney U test was used to assess the disparities in the power spectrum of the frequency bands between the hypothermia and the control arms. The three SD segments in the hypothermia group were compared with their counterparts in the control group. After the U-Mann Whitney test, the Holm-Bonferroni adjustment was applied to address multiple comparisons across time (*p* = 0.05/3). Consequently, the revised value of *p* was established at 0.017. The outcomes depicted in Figure 6; Supplementary Table 6 are those with value of *p* less than 0.0017 in the overall iteration of the U-Mann Whitney test.

In the figures and tables, the *p*-value in both statistical analyses has the following notation: * is equal to ≤0.05; ** is equal to ≤0.01; *** is equal to ≤0.001.

## 3 Results

Six subjects were used in the control arm. The ECoG recording time in the control group was 20 h in three subjects, 19 h, and 17 h in the last two experiments. Data from one control animal was excluded due to the lack of arterial occlusion corroborated in the postmortem exploration. Hypothermia was applied in 6 subjects. The recording time was 20 h in four experiments and 19 h in the last two tests.

To prove the reliability of the power spectra analysis and the reasonable variability of the data in the infarct evolution analysis, the 5 min signal segments recorded before MCAo of all the subjects from all the groups were analyzed by applying the Kruskal-Wallis test. No statistically differences were reported (Figures 1–5; Supplementary Tables 1–5).

### 3.1 Changes in frequency bands in the insulted and healthy hemispheres under normothermic and hypothermic conditions

The output reported in this section and further discussed is based on the analysis of the differences in the power of the frequency bands within the 4 conditions for each timepoint. To observe the alterations of the power of each frequency band over time in each separate condition, please refer to Supplementary Figures 1–4; Supplementary Tables 7–10.

#### 3.1.1 Insulted hemisphere under normothermia

The power spectrum of the five frequency bands was stable in the insulted hemisphere under normothermia (red bars in Figures 1–5 and red slots in Supplementary Tables 1–5) and predominantly stayed unchanged in E1 and E2. The delta band displayed steady values between the 8th and 12th hour after MCAo in E1 and E2 (Figure 1; Supplementary Table 1). Theta activity was notable at 8th hour in E1 and E2 (Figure 2; Supplementary Table 2). The alpha band was prominent at the 8th hour in E1 (Figure 3; Supplementary Table 3), whereas the beta showed heightened activity between the 4th and 8th hour in E1 and at the 16th hour in E2 (Figure 4; Supplementary Table 4). The power of gamma was persistent from the 8th to the 12th hour in E1 and between the 8th and 16th hour in E2 (Figure 5; Supplementary Table 5). Only in E4 was the same effect exceptionally observed in the beta band, where the power spectrum of beta was sustained and elevated from the 4th to the 16th hour (Figure 4; Supplementary Table 4).

Conversely, the power spectra of the five frequencies experienced a power decline in E3, E4, and E5 from the early stages of the ECoG recording. The delta band showed decreased activity from the 8th to the 16th hour in E3, and right after the MCAo to the 16th hour in E4 and E5 (Figure 1; Supplementary Table 1). Theta activity was depressed within the 4th and 8th hour in E3, between the 4th and 8th hour in E4, and within the 4th and 12th in E5 (Figure 2; Supplementary Table 2). The power of the alpha wave diminished at the 4th hour in E5 (Figure 3; Supplementary Table 3). The beta band decayed right after the MCAo in E4, right after the vascular occlusion to the 12th hour in E5, and up to the 16th hour in E3 (Figure 4; Supplementary Table 4). Finally, gamma activity was lessened immediately after the MCAo to the 8th hour in E3, at the 8th hour in E4, and within the 4th and 12th hour in E5 (Figure 5; Supplementary Table 5). Similar patterns were reported in E1 and E2, but in the late stages of the ECoG recording, such as at the 16th hour in E1 and E2 for the delta band (Figure 1; Supplementary Table 1) and only in E2 for alpha activity (Figure 3; Supplementary Table 3).

#### 3.1.2 Healthy hemisphere under normothermia and hypothermia

In normothermic healthy hemispheres (yellow bars in Figures 1–5 and yellow slots in Supplementary Tables 1–5), no modifications in the power spectrum of the five frequency bands were observed over the experiments and across the comparison within the other three conditions (Figures 1–5; Supplementary Tables 1–5).

For the healthy hemisphere under hypothermia (blue bars in Figures 1–5 and blue slots in Supplementary Tables 1–5) the values of the power spectrum of delta remained elevated at the 8th hour after the MCAo in E1 and E2, from the 4th th to 8th hour in E4 and E5, and within the 8th the 12th hour in E3 (Figure 1; Supplementary Table 1). The same findings were observed in theta band at the 4th hour in E3 to E5. However, the theta band exhibited a power decay from the 12th to the 16th hour in E1, and at the 16th hour in E2 to E5 (Figure 2; Supplementary Table 2). For the fast frequencies, the three consistently suffered a power collapse. The alpha wave was depressed from the 8th to the 16th hour at E1–E4, and within the 12th and 16th hour in E5 (Figure 3; Supplementary Table 3), while the beta band showed a decline between the 12th and 16th hour in E1, and at the 16th hour in E3 to E5 (Figure 4; Supplementary Table 4). The gamma band demonstrated decreased power from the 8th to 16th hour in E1 and E2, and between the 12th and 16th hour in E3 and E5 (Figure 5; Supplementary Table 5).

#### 3.1.3 Insulted hemisphere under hypothermia

The five frequency bands and related power spectra were consistently depressed under hypothermia regardless of the electrode location in the insulted hemisphere (purple bars in Figures 1–5 and purple slots in Supplementary Tables 1–5). The power spectra depression was constantly observed after the complete establishment of mild hypothermia at the 4th hour after MCAo. Delta underwent a power decline from the 8th hour after the MCAo to the 16th hour in E1 to E3, and from the 4th to the 16th hour in E4 and E5 (except at the 8th hour in E4) (Figure 1; Supplementary Table 1). Theta band encountered power reductions from the 4th to the 12th hour in E5, from the 4th to the 16th hour in E4, and between the 8th and the 16th hour in E1 to E3 (except at the 12th hour in E2) (Figure 2; Supplementary Table 2). Alpha activity was reduced from the 8th, and 16th hour in E4, and between the 12th and 16th hour in E5 (Figure 3; Supplementary Table 3). The beta band experienced a power spectrum fall from the 4th to the 12th hour in E1, within the 4th and the 16th hour in E2, E4, and E5, and between the 8th and the 12th hour in E3 (Figure 4; Supplementary Table 4). The gamma wave was depressed at the 4th hour in E5, between the 8th and 12th in E3, and at the 8th and 16th hour in E1 and E2 (Figure 5; Supplementary Table 5).

### 3.2 Changes in frequency bands before, during, and after the SD formation in the insulted hemisphere under normothermic and hypothermic conditions

Eighty-two SDs were recorded in the hypothermia arm, whereas 219 were from the normothermia group. No SDs were recorded in the right healthy hemispheres. For the power spectra analysis, 27 SDs from the E1L, 14 SDs from E2L, and 13 SDs from E3L were used. The analyzed SDs were collected from the ECoG recording after the 4th hour of the MCAo, where the mild hypothermia was completely established at 32°C. To avoid any summation effect in the power spectrum analysis, the same amount of SDs were used from the normothermia group for the comparison with the hypothermia arm: 27 SDs from E1L, 14 SDs from E2L, and 13 SDs from E3L. The SDs from the normothermia group were also obtained from the same ECoG timeframe, from the 4th hour onwards. All the SDs used in the power spectrum analysis had neither artifact in 5-min signal segments before and after the SD development nor other SDs.

In E1L, the hypothermia group exhibited elevated power spectra in the delta during the SD, and postSD phases, as well as beta in the three SD intervals, surpassing those of the normothermia arm. Within E2L, the hypothermia group maintained higher beta activity values than the normothermia arm in the preSD and postSD intervals, while delta activity was more elevated only in the preSD phase. In E3L, the hypothermia group demonstrated higher beta band values compared to the normothermia arm in the postSD phase. However, the normothermia group displayed more exalted power spectra in the delta, theta, and alpha bands during the preSD, SD, and postSD phases than the hypothermia arm (Figure 6; Supplementary Table 6).

## 4 Discussion

In our present work, we could identify the modifications experienced by the frequency bands according to the perfusion status and temperature of the brain hemispheres. The insights obtained from the insulted hemisphere in normothermia support the power spectra patterns of the frequency bands observed in our first study regarding the vascular occlusion and the electrode location (Kentar et al., 2022). The electrodes close to the ischemic core (E3 to E5) registered power spectra decline in all the frequency bands in the early stages of the infarct evolution. In contrast, the further ones in E1 and E2 reported the persistence of the five frequencies until their power decay took place in the late phase of the ECoG recording.

Furthermore, we investigated the neurophysiological characteristics of mild hypothermia in both normal brain tissue and during an ischemic stroke. In the healthy hemisphere, the power spectra of delta and theta bands persisted elevated even when mild hypothermia was completely established at 32°C. Conversely, the fast frequencies were depressed upon rapidly reaching lower temperatures.

For the insulted hemispheres under hypothermia, the depression of the frequency bands was consistently reported after the 4th hour of the MCAo, once mild hypothermia had been fully implemented at 32°C regardless of the cortical location. We demonstrated that mild hypothermia promoted the attenuation of the frequency bands, indicating the suppression of brain activity, which could account for the reduced energy demand and the previously observed shrinkage in infarct volume (Kentar et al., 2023).

In our former work, we proved that mild hypothermia considerably reduces the incidence and expansion of SD (Kentar et al., 2023). A novel finding from our research regarding mild hypothermia is that electrodes situated distant from the ischemic core in E1 and E2 exhibited improved preservation of frequency bands at lower temperatures. In both electrodes, mild hypothermia maintained the power spectra of frequency bands more effectively before the SD formation of spreading depolarization (preSD), prevented their decline in power during SD development, and supported their recovery after the SD migration (postSD). Unfortunately, the electrode close to the ischemic core in E3 was less benefited by the mild hypothermia.

It is also pertinent to consider the effects of anesthetics on frequency bands when assessing their resulting power spectra. Midazolam, propofol, and isoflurane induce an unconscious state by lessening the fast frequencies and promoting the slower bands. In addition, one of the main anatomical targets of the anesthetic agents we used is the frontoparietal cortex, our region of interest. Thus, anesthetic drugs might have exerted an influence on the basal power spectrum of the frequency bands in our ECoG recordings (Akeson et al., 1993; Makiranta et al., 2002; Bojak et al., 2015; Mirra et al., 2022). On the other hand, anesthetic drugs such as isoflurane can also impede the onset of SD development, resulting in a reduction of SD occurrence in all experimental scenarios (Takagaki et al., 2014; Klass et al., 2018).

### 4.1 Frequency bands are depressed early in the electrodes near the MCAo and late in the distant electrodes in the insulted hemisphere under normothermia

As proposed in our core-penumbra map (Kentar et al., 2022), the electrodes closest to the infarcted area recorded an early drop in the spectral power of frequency bands. As the electrodes moved away from the blood flow-deprived zone, the power depression of frequency bands was observed later, concurring with the results previously described in EEG (Rabiller et al., 2015).

The premature power slumps of the frequency bands were observed in E5, followed by E4. In E4 and E5, both slow and fast frequencies decayed right after the vascular clamping or at the 8th hour at the latest, as observed in gamma at E4. The power depression in E4 and E5 remained until the 16th hour, reporting no power recovery in E5 and almost in E4. Nevertheless, there were power fluctuations in the beta band within E4. The beta band exhibited a decrease in power immediately after MCAo, but it subsequently underwent a recovery that persisted until the final timepoint (Figure 4; Supplementary Table 4). In our previous study, we detected the same trend in electrodes E2L and E4L (Kentar et al., 2022). The heterogeneous progression of infarct development could have an impact on the beta band fluctuations observed in our current work in E4 and in E2L and E4L in our previous study (Dereski et al., 1993; del Zoppo et al., 2011; Guo et al., 2014; Khan et al., 2020).

E3 exhibited a similar pattern as E4 and E5 with early suppression of beta and gamma at 0th hour (Figures 4, 5; Supplementary Tables 4, 5). However, delta, theta, and alpha bands persisted until the 4th hour after MCAo. At that point, theta experienced a significant power decline with no potential for recovery, observing the same trend by delta and theta with no statistically significant outputs. (Figures 1–3; Supplementary Tables 1–3). We are convinced that the E3 is in the penumbra area, where the stunned cells remain partially active, maintaining alpha and slow frequencies for 4 h. Beyond the 4th hour, the cerebral blood flow in the penumbra decreases below the infarct threshold, and the stunned cells die, becoming part of the ischemic core and losing all the frequency bands in the process (Rabiller et al., 2015). Therefore, we support other authors while using alpha and delta bands for the estimation of brain perfusion in ischemic scenarios, such as delayed cerebral ischemia (Gollwitzer et al., 2015; Foreman et al., 2018; Rosenthal et al., 2018; Zheng et al., 2022).

Finally, E1 and E2, located distant to the ischemic core, registered maintained activity of the five frequency bands up to the 12th hour after the MCAo. All the frequency bands fell during the 12th and 16th hour, with the most significant slumps observed at the 16th hour in the delta band in both electrodes and alpha in E2 (Figures 1, 3; Supplementary Tables 1, 3). At the late stage of the stroke, the infarction and the secondary damage caused by the SDs reached the distant cortex under E1 and E2, provoking the depression of the frequency bands (Hartings et al., 2009, 2020).

### 4.2 Slow frequencies endured upon the establishment of mild hypothermia, while the fast frequencies were entirely suppressed in the healthy hemisphere

After lowering the brain temperature to around 32°C at the 4th hour after MCAo, the healthy hemisphere experienced the collapse of all the fast frequencies, irrespective of the electrode location. Their power exhibited a significant decline, starting with alpha from the 8th hour, followed by gamma between the 8th and 12th hour, and finally beta between the 12th and 16th hour (Figures 3–5; Supplementary Tables 3–5). In contrast, the power of slow frequency bands remained consistently high upon reaching mild hypothermia at the 4th hour, with the delta band being the predominant one until the 8th hour across all recorded brain tissue (Figure 1, Supplementary Table 1). A similar pattern was observed in the theta band but was limited to a brief period at the 4th hour in E3 to E5. Subsequently, theta activity significantly declined across all electrodes between the 12th and 16th hour (Figure 2; Supplementary Table 2).

Our results coincide with the physiological measurements reported using non-invasive monitoring, which document a mismatching between slow and fast frequency bands, namely the theta and beta bands at a temperature of 33.5°C, resembling the rhythmic patterns observed during non-REM sleep. Furthermore, we noted that at the 16th hour, while maintaining a brain temperature of approximately 32°C, all frequency bands experienced power suppression. The generalized power collapse could be consistent with the initiation of a deceleration in the remaining frequencies, eventually leading to an isoelectric signal observed in more profound hypothermia at 25°C (Pearcy and Virtue, 1960; Deboer and Tobler, 1995; Kochs, 1995; Abend et al., 2009; Mahfooz et al., 2017; Myers et al., 2017).

### 4.3 Mild hypothermia depressed all the frequency bands in the insulted hemisphere

The power suppression induced by the full onset of mild hypothermia after the 4th hour was also documented in the insult hemisphere. Fast frequencies displayed power depression, which was more significant in E5 by the alpha and gamma (Figure 3; Supplementary Table 3), and across all the electrodes in beta band after the 4th hour onwards (Figures 4, 5; Supplementary Tables 4, 5). In contrast to the healthy hemisphere under hypothermia, the dominance of the slow frequency bands was absent in ischemic scenarios. Consequently, the power decay in slow frequencies became evident, occurring in the early stages of the delta and theta bands from the 4th hour in electrodes E4 and E5, and after the 8th hour in electrodes E1 to E3 (Figures 1, 2; Supplementary Tables 1, 2).

During the ischemic process, the metabolism shifts to anaerobic, culminating in intracellular acidosis, ion gradient collapse, intracellular edema, and eventually neuronal death (Sun et al., 2019). Mild hypothermia prevents the loss of essential metabolic substrates, preserves high-energy phosphate compounds, and maintains tissue pH by reducing neuronal metabolic demands (Kurisu and Yenari, 2018). The attenuation of power in frequency bands serves as a tangible representation of the reduction of the basal activity of the brain induced by mild hypothermia (Pearcy and Virtue, 1960; Kochs, 1995; Abend et al., 2009; Mahfooz et al., 2017; Myers et al., 2017). The decreased metabolic demands reduce the neuronal vulnerability to death provoked by the lack of blood flow and metabolites. Consequently, mild hypothermia sustains a constricted infarct zone and hinders the expansion of the ischemic core (Colbourne et al., 2000; Kollmar et al., 2007; van der Worp et al., 2007; Zhang et al., 2007; Clark et al., 2008; Kentar et al., 2023).

It is evident that both insulted hemispheres, whether under normothermia or hypothermia, experienced power depression. There are no discernible differences in the power decay over time in E3 to E5 between the two conditions, as the brain infarct alters the power spectra. However, variations in the power decay are more pronounced temporally in regions farther from the ischemic core, such as in E1 and E2. For example, the reduction in power in the delta band was reported earlier at the 8th hour in both electrodes in the insulted hemisphere under hypothermia compared to the power decline observed in the insulted hemisphere under normothermia at the 16th hour (Figure 1; Supplementary Table 1). Likewise, the reductions in theta activity occurred at the 8th hour under hypothermia and at the 12th hour under normothermia (Figure 2; Supplementary Table 2). The beta band declined at the 4th hour under hypothermia and between the 8th and 12th hour under normothermia (Figure 4; Supplementary Table 4), while gamma activity decayed at the 8th hour under hypothermia, and it was sustained with minor reductions under normothermia (Figure 5; Supplementary Table 5).

As predicted, the early decrease in power spectrum across various frequency bands in non-vascular compromise brain tissue, like in E1 and E2, shows reduced brain activity linked to a decline in brain temperature. Therefore, employing the frequency bands as biomarkers can guide the effective application of mild hypothermia while minimizing the adverse effects of excessively low temperatures. But before establishing frequency bands as biomarkers for mild hypothermia application, it is crucial to investigate a comprehensive association between the changes in the power of the frequency bands and the modifications in the brain temperature completely. The rewarming effect in the vascular insulted cortex remains an unresolved issue, and future studies should be focused on determining whether the rise in brain temperature is related to the restoration of the power of frequency bands (Pearcy and Virtue, 1960; Levy, 1984; Shin et al., 2006).

### 4.4 Mild hypothermia maintained considerably the power spectra of the frequency bands before, during, and after the SD formation in electrodes distant from the ischemic core

Mild hypothermia displayed significant preservation of power spectra the further the electrodes from the ischemic core are. The brain tissue beneath E1L experienced the most advantages, showing increased power spectra in the delta during the SD and postSD periods, and in beta band across all three SD phases. Afterward, E2L was the subsequent electrode that registered better preservation of the power spectrum in the beta band, but only before and after the SD formation, and in the delta band in the preSD phase. In E3, just the beta band during the postSD displayed a high power, while the power of slow frequency bands and alpha significantly decreased under lower temperatures (Figure 6; Supplementary Table 6).

In our previous work, we demonstrated that mild hypothermia reduced the occurrence and expansion of SDs (Kentar et al., 2023), concurring with past studies about the relationship between brain temperature and SD formation (Sasaki et al., 2009; Schiefecker et al., 2018). Based on the presented results, we confirm that the brain activity represented by the frequency bands is better maintained under cold temperatures prior to, during, and after SDs. However, mild hypothermia is more effective in brain tissue with minimal or null vascular compromise, as observed in E1L and E2L, where the neural net remains intact.

Conversely, brain tissue that is vascular compromised, such as the penumbra, proves to be less benefited by the mild hypothermia, as witnessed in E3L. In the penumbra, the neural net dysfunction and the dysregulation of ion gradients may occur prior to reaching 32°C, resulting in a subsequent formation of SD and cyclic damage of the neuronal net (Dreier and Reiffurth, 2015; Kramer et al., 2016; Hartings et al., 2017). The quick solution might be to strengthen the hypothermia application or establish it early and for a long time to nullify any possible brain excitability. Nevertheless, the incidence of adverse effects and complications is greater the more the temperature is lowered (Hypothermia after Cardiac Arrest Study Group, 2002; Shankaran et al., 2005; Azzopardi et al., 2009; Tsuda et al., 2017).

### 4.5 Future directions

Our forthcoming projects will tackle two key issues: Recanalization and collateral flow. Hypothermia plus recanalization reduces infarct zone volume, mitigates cerebral edema, prevents hemorrhagic transformation, and improves neurological outcome (Hong et al., 2014; Mattingly et al., 2016; Hwang et al., 2017; Lee and Ding, 2020). However, it is unclear how electrical activity in injured brains will be re-established after mild hypothermia and vascular recanalization.

A good collateral flow can overcome the harsh ischemic environment in injured cortical and subcortical zones by compensating for the blood supply restriction (Bang et al., 2015; Cuccione et al., 2016; Ma et al., 2020). The motivation to study the collateral flow lies in exploring the variation in infarct evolution considering anatomical and neurophysiological aspects within ipsilateral regions, hemispheres, and even subjects.

There is no consensus regarding the appropriate application of hypothermia as a therapeutic strategy in stroke. The optimal time for cooling, the doses, and the time window might be personalized according to biomarkers (Lyden, 2020). Currently, the recommendation is to establish therapeutic hypothermia at 33–34°C, extend the time window for therapy initiation to obtain more promising results, decrease the treatment duration to minimize any adverse effect, and apply less invasive cold therapies for pharmacological and non-pharmacological interventions that directly affect the brain temperature (Meloni et al., 2008; Yenari and Hemmen, 2010; Dumitrascu et al., 2016). The main objective is to support, with compelling scientific evidence, the proper use of mild hypothermia in stroke, as well as to ensure the feasibility of its clinical application to establish it as a standard therapy. Nevertheless, all these recommendations should be verified through experiments, and animal models are suitable for testing their effectiveness.

Regarding the frequency band analysis, recent findings have demonstrated its effectiveness in anticipating SD formation in patients with traumatic brain injury, highlighting delta as the most sensitive band among the others for discerning SD occurrence (Chamanzar et al., 2023). The relevance of delta band in the frequency band analysis was first approached in the work of Hartings and cols. The delta activity decay was associated with the temporally isolated, transient depressions of spontaneous EEG amplitudes in the vascular insulted hemisphere caused by the SD occurrence (Hartings et al., 2014). Based on the results of both studies and our findings, we aim to extend our understanding by exploring how delta band respond timewise and locally in the vascular insulted cortex, in the context of mild hypothermia and other potential neuroprotective interventions. We want to prove whether variations in the delta band not only show correlations with the SD occurrence but also serve as a metric for evaluating the efficacy of neuroprotective interventions.

Another interesting topic for investigation involves the phenomenon known as partial superficial SDs, which exhibited distinct modifications in frequency bands compared to those observed in fully propagated SDs. Recently, it was revealed persistence of the delta band during partial superficial SDs, in contrast to its complete suppression during fully propagated SDs (Nasretdinov et al., 2023). While our findings align with those of fully propagated SDs (Kentar et al., 2022), the existence of partial SDs introduces the possibility of new patterns of alterations in frequency bands induced by these events. We also posit a hypothesis suggesting that partial SDs may exhibit a more favorable response to mild hypothermia compared to fully propagated ones, although this remains a subject for future exploration on our side.

## Data availability statement

The datasets presented in this study can be found in online repositories. The names of the repository/repositories and accession number(s) can be found at: Diaz Peregrino, Roberto (2023), “SD_Power_Spectra_Hypothermia_control”, Mendeley Data, V1, doi: 10.17632/x9fhdy6tbt.1; Diaz Peregrino, Roberto (2023), “Infarct_progression_Power_Spectra_Hypothermia_control”, Mendeley Data, V1, doi: 10.17632/twrnv93y82.1, https://data.mendeley.com/datasets/twrnv93y82.

## Ethics statement

The animal study was approved by Institutional Animal Care and Use Committee in Karlsruhe, Baden-Württemberg. The study was conducted in accordance with the local legislation and institutional requirements.

## Author contributions

RD-P: Data curation, Formal analysis, Investigation, Methodology, Writing – original draft, Writing – review & editing. MK: Data curation, Formal analysis, Investigation, Methodology, Writing – original draft, Writing – review & editing. CT: Formal analysis, Methodology, Writing – original draft, Writing – review & editing. RS-P: Methodology, Supervision, Writing – review & editing. PA-P: Methodology, Resources, Writing – review & editing. FR-C: Writing – review & editing. DS-J: Writing – review & editing. AU: Writing – review & editing. JW: Writing – review & editing. ES: Conceptualization, Funding acquisition, Supervision, Validation, Visualization, Writing – review & editing.
