# Supplementary material for: The neurophysiological effect of mild hypothermia in gyrencephalic brains submitted to ischemic stroke and spreading depolarizations
Source: Front Neurosci. 2024 Mar 14;18:1302767. doi: 10.3389/fnins.2024.1302767 (PMC10986791; doi:10.3389/fnins.2024.1302767)
Supplement: Supplementary file 1 [file Data_Sheet_1.PDF]

DELTA

Pre-MCAo

Exp	Group	M=	SD=
E1	KKW	χ2 = 0.601	p = 0.896
E1	1	10.5586	3.5006
E1	2	10.4682	5.5663
E1	3	11.3482	1.6941
E1	4	11.7717	3.1128
E2	KKW	χ2 = 2.884	p = 0.410
E2	1	10.9628	2.8408
E2	2	9.2506	3.1210
E2	3	11.5612	2.1134
E2	4	11.5968	1.6497
E3	KKW	χ2 = 6.213	p = 0.102
E3	1	7.9348	2.6349
E3	2	10.0810	4.7992
E3	3	11.7882	1.9443
E3	4	12.0628	2.6900
E4	KKW	χ2 = 3.004	p = 0.391
E4	1	8.4142	4.7024
E4	2	12.3094	2.5814
E4	3	11.4932	1.6979
E4	4	11.5200	3.4648
E5	KKW	χ2 = 7.243	p = 0.065
E5	1	8.0968	3.8627
E5	2	15.2946	3.5788
E5	3	11.3087	2.2322
E5	4	10.3720	3.8115

0h

Exp	Group	M=	SD=	Dunn´s test 1	2	3	4
E1	KKW	χ2 = 1.071	p = 0.784				
E1	1	10.6632	2.5192				
E1	2	10.0068	5.0480				
E1	3	10.4710	1.8773				
E1	4	11.6402	2.3445				
E2	KKW	χ2 = 1.464	p = 0.691				
E2	1	8.9220	3.7554				
E2	2	8.0172	3.6062				
E2	3	10.8065	2.0780				
E2	4	9.5233	4.2670				
E3	KKW	χ2 = 2.247	p = 0.523				
E3	1	8.7956	3.7822				
E3	2	9.1466	3.1421				
E3	3	11.4600	1.4588				
E3	4	10.2143	3.3978				
E4	KKW	χ2 = 11.949	**p = 0.008**				
E4	1	8.8809	4.4627				
E4	2	3.7782	3.3659			*	
E4	3	10.9417	1.3767		*		*
E4	4	3.6485	2.8508			*	
E5	KKW	χ2 = 12.257	**p = 0.007**				
E5	1	9.0343	3.1846				
E5	2	7.0786	3.3708				
E5	3	11.0185	1.7061				**
E5	4	3.1838	2.1848			**	

4h

Exp	Group	M=	SD=	Dunn´s test 1	2	3	4
E1	KKW	χ2 = 0.216	p = 0.975				
E1	1	9.8976	4.4400				
E1	2	10.1638	4.0827				
E1	3	10.8623	1.8926				
E1	4	10.6383	2.0129				
E2	KKW	χ2 = 3.828	p = 0.281				
E2	1	8.6064	3.4064				
E2	2	7.8132	1.7118				
E2	3	11.0110	1.8981				
E2	4	9.2333	3.4007				
E3	KKW	χ2 = 11.254	p = 0.010				
E3	1	9.7454	4.2082				
E3	2	1.6598	0.4796			*	
E3	3	11.0258	1.6873		*		
E3	4	9.7148	3.6344				
E4	KKW	χ2 = 15.204	**p = 0.002**				
E4	1	8.5548	3.8701				
E4	2	2.0844	1.8004			*	
E4	3	10.7275	1.6443		*		**
E4	4	1.7558	1.4850			**	
E5	KKW	χ2 = 15.534	**p = 0.001**				
E5	1	10.4388	3.9864				*
E5	2	2.0646	2.2009				
E5	3	10.7923	1.4939				*
E5	4	0.9603	0.8591	*		*	

8h

Exp	Group	M=	SD=	Dunn´s test 1	2	3	4
E1	KKW	χ2 = 12.613	**p = 0.006**				
E1	1	10.3916	3.6582				*
E1	2	9.8360	3.4844				*
E1	3	9.5560	1.1093				*
E1	4	1.3690	1.1380	*	*	*	
E2	KKW	χ2 = 15.028	**p = 0.002**				
E2	1	10.5652	2.2568				**
E2	2	7.7328	1.8217				
E2	3	9.8495	1.2922				**
E2	4	1.1662	0.9256	**		**	
E3	KKW	χ2 = 16	**p = 0.001**				
E3	1	8.9376	2.6296				
E3	2	1.1514	0.2865			*	
E3	3	9.6347	1.1530		*		**
E3	4	1.4415	1.2442			**	
E4	KKW	χ2 = 16.835	**p = 0.001**				
E4	1	8.4774	4.3598		**		
E4	2	0.7024	0.5184	**		**	
E4	3	8.9727	1.0301		**		
E4	4	1.3757	0.8257				
E5	KKW	χ2 = 15.286	**p = 0.002**				
E5	1	8.1809	4.2155		*		*
E5	2	1.2094	1.9767	*		*	
E5	3	8.8525	1.1638		*		*
E5	4	0.7150	0.5914	*		*	

12h

Exp	Group	M=	SD=	Dunn´s test 1	2	3	4
E1	KKW	χ2 = 15.699	**p = 0.001**				
E1	1	10.2892	2.9293				**
E1	2	8.8888	3.3584				**
E1	3	5.7725	1.1662				
E1	4	1.1992	0.8543	**	**		
E2	KKW	χ2 = 14.957	**p = 0.002**				
E2	1	9.1022	3.7774				**
E2	2	7.4852	2.0187				*
E2	3	5.1592	0.8626				
E2	4	0.9467	0.9906	**	*		
E3	KKW	χ2 = 16.426	**p = 0.001**				
E3	1	7.4610	2.9405		**		*
E3	2	0.7356	0.1397	**		*	
E3	3	5.3612	0.6652		*		
E3	4	1.2403	1.0194	*			
E4	KKW	χ2 = 16.348	**p = 0.001**				
E4	1	8.0776	3.6556		*		**
E4	2	0.6132	0.3475	*			
E4	3	5.2567	0.9729				
E4	4	0.6517	0.3775	**			
E5	KKW	χ2 = 15.454	**p = 0.001**				
E5	1	9.3046	4.2503		*		*
E5	2	1.0596	1.7971	*			
E5	3	5.5450	1.0020				
E5	4	0.5788	0.4586	*			

16h

Exp	Group	M=	SD=	Dunn´s test 1	2	3	4
E1	KKW	χ2 = 16.645	**p = 0.001**				
E1	1	9.8078	3.4143		*		***
E1	2	1.6788	1.0266	*			
E1	3	2.7745	0.7001				
E1	4	0.8173	0.6396	***			
E2	KKW	χ2 = 16.747	**p = 0.001**				
E2	1	9.9288	2.8360		*		***
E2	2	1.2540	0.8154	*			
E2	3	2.8643	0.6718				
E2	4	0.8203	0.9623	***			
E3	KKW	χ2 = 15.563	**p = 0.001**				
E3	1	10.0010	3.4417		**		**
E3	2	0.5426	0.1170	**			
E3	3	2.3927	0.5384				
E3	4	0.8180	1.3485	**			
E4	KKW	χ2 = 17.363	**p = 0.001**				
E4	1	9.2166	4.3020		**		**
E4	2	0.4476	0.2053	**			
E4	3	2.1950	0.7380				
E4	4	0.5077	0.4140	**			
E5	KKW	χ2 = 14.314	**p = 0.003**				
E5	1	8.9750	4.6658		*		**
E5	2	1.1092	1.1773	*			
E5	3	1.8100	0.9538				
E5	4	0.7425	0.2568	**			

Supplementary Table 1. Power spectra of the delta band in the insulted and healthy hemispheres under normothermic and hypothermic conditions. The Kruskal-Wallis test was computed to perform the multiple comparisons within the 4 conditions: (1) the healthy hemisphere under normothermia (1), the insulted hemisphere under normothermia (2), the healthy hemisphere under hypothermia (3), and the insulted hemisphere under hypothermia (4). Dunn´s test was executed to determine which specific subanalyses revealed statistically significant differences. Both analyses were applied at 0h, 4h, 8h, 12h, and 16h. After Dunn´s test estimations, a Holm-Bonferroni adjustment was performed for multiple comparisons over time (p =0.05/6). Therefore, the adjusted p-value was set at 0.0083. The results from Dunn´s test shown in Figures 1-5 are the ones with p-values below 0.0083 in the general iteration of the Kruskal Wallis test (**p-values underlined and bold**). In Dunn´s test, the statistically significant changes in the power spectrum of the frequency bands within conditions are represented as * when the p-value was equal to ≤0.05, ** when it was equal to ≤0.01, and as *** when it was equal to ≤0.001. KKW, Kruskal-Wallis test; M, mean; SD, standard deviation, χ2, chi-square.

THETA

Pre-MCAo

	KKW			M	SD
E1	$\chi 2$ = 1.483	p = 0.686	1	5.1672	2.4523
			2	6.1898	2.3230
			3	6.0997	1.8408
			4	5.0202	1.6638
E2	$\chi 2$ = 1.417	p = 0.701	1	4.9680	1.9609
			2	4.3120	2.6088
			3	5.7457	1.2341
			4	4.7170	1.9234
E3	$\chi 2$ = 4.3	p = 0.231	1	4.8920	2.7108
			2	4.5930	1.1382
			3	6.1647	1.0469
			4	4.6558	1.8585
E4	$\chi 2$ = 2.874	p = 0.412	1	4.6786	1.7047
			2	5.8200	2.1530
			3	6.1687	0.8205
			4	5.2928	2.0406
E5	$\chi 2$ = 4.939	p = 0.176	1	4.1782	1.7251
			2	7.0586	2.1950
			3	5.6583	0.9319
			4	5.0803	2.3683

0h

	KKW			M	SD	Dunn´s test 1	2	3	4
E1	$\chi 2$ = 3.270	p = 0.352	1	4.1452	1.5947				
			2	5.6374	2.6932				
			3	5.7672	1.9593				
			4	3.9763	2.0358				
E2	$\chi 2$ = 5.429	p = 0.143	1	3.7844	2.2311				
			2	4.1374	1.8495				
			3	5.8085	1.0330				
			4	3.6497	1.8614				
E3	$\chi 2$ = 7.655	p = 0.054	1	3.0800	2.7246				
			2	4.0644	1.1613				
			3	5.8688	0.9917				
			4	3.8293	1.2479				
E4	$\chi 2$ = 11.678	p = 0.009	1	4.8710	1.9938				
			2	1.9146	1.2094			*	
			3	5.5432	0.9439		*		
			4	2.7698	1.1853				
E5	$\chi 2$ = 10.572	p = 0.014	1	5.1874	1.9118				
			2	3.3922	1.3011				
			3	5.8065	0.6864				*
			4	3.1733	1.3936			*	

4h

	KKW			M	SD	Dunn´s test 1	2	3	4
E1	$\chi 2$ = 10.555	p = 0.014	1	3.8146	2.5586				
			2	5.6858	1.9629				**
			3	3.9103	1.1614				
			4	1.2312	1.2781		**		
E2	$\chi 2$ = 7.858	p = 0.049	1	4.2106	3.0136				
			2	3.7702	1.6179				
			3	3.3545	0.7756				
			4	1.3097	1.2123				
E3	$\chi 2$ = 12.549	**p = 0.006**	1	3.3684	2.5907				
			2	0.4668	0.3300			*	
			3	4.5803	0.4810		*		
			4	1.2933	1.3283				
E4	$\chi 2$ = 14.470	**p = 0.002**	1	4.4386	3.2006				
			2	0.5980	0.2395			*	
			3	4.4592	0.7252		*		*
			4	0.9542	0.7966			*	
E5	$\chi 2$ = 16.029	**p = 0.001**	1	4.3914	1.9756				
			2	0.4640	0.2900			*	
			3	4.5615	0.5928		*		**
			4	0.7157	0.8308			**	

8h

	KKW			M	SD	Dunn´s test 1	2	3	4
E1	$\chi 2$ = 16.179	**p = 0.001**	1	4.3436	2.6053				*
			2	5.1074	1.8544				**
			3	1.0153	0.2508				
			4	0.5877	0.5609	*	**		
E2	$\chi 2$ = 17.223	**p = 0.001**	1	3.9600	2.3673				**
			2	3.4996	1.2234				**
			3	0.8948	0.2065				
			4	0.5620	0.1580	**	**		
E3	$\chi 2$ = 14.763	**p = 0.002**	1	4.5006	1.9110		**		**
			2	0.4546	0.3149	**			
			3	1.0365	0.2490				
			4	0.4905	0.5333	**			
E4	$\chi 2$ = 14.336	**p = 0.002**	1	4.7244	1.5643		**		**
			2	0.4930	0.2667	**			
			3	1.0110	0.2925				
			4	0.5473	0.4166	**			
E5	$\chi 2$ = 15.043	**p = 0.002**	1	4.5038	1.4259		**		*
			2	0.4574	0.2246	**			
			3	0.9745	0.1719				
			4	0.6293	0.3394	*			

12h

	KKW			M	SD	Dunn´s test 1	2	3	4
E1	$\chi 2$ = 12.348	**p = 0.006**	1	5.3484	2.0649			*	*
			2	1.7166	1.2233				
			3	0.5753	0.1063	*			
			4	0.6072	0.4581	*			
E2	$\chi 2$ = 11.576	p = 0.009	1	4.4982	2.5399			*	*
			2	0.6052	0.2755				
			3	0.5508	0.0891	*			
			4	0.4798	0.3042	*			
E3	$\chi 2$ = 11.754	**p = 0.008**	1	3.4040	1.0810				**
			2	0.4920	0.3062				
			3	0.4700	0.0944				
			4	0.4293	0.3633	**			
E4	$\chi 2$ = 14.269	**p = 0.003**	1	4.6758	2.6902				***
			2	0.5038	0.2392				
			3	0.5968	0.1461				
			4	0.3133	0.2323	***			
E5	$\chi 2$ = 12.655	**p = 0.005**	1	4.2572	1.7387		*		**
			2	0.3494	0.2084	*			
			3	0.5187	0.1117				
			4	0.4095	0.3403	**			

16h

	KKW			M	SD	Dunn´s test 1	2	3	4
E1	$\chi 2$ = 15.712	**p = 0.001**	1	4.5386	1.9876			***	
			2	0.9498	0.7129				
			3	0.1842	0.0630	***			
			4	0.4945	0.3192				
E2	$\chi 2$ = 12.460	**p = 0.006**	1	3.9340	2.2098			**	*
			2	0.4384	0.3564				
			3	0.1867	0.0354	**			
			4	0.2785	0.2278	*			
E3	$\chi 2$ = 15.986	**p = 0.001**	1	3.8998	1.8182			***	*
			2	0.4792	0.2166				
			3	0.1793	0.0462	***			
			4	0.3507	0.2418	*			
E4	$\chi 2$ = 12.319	**p = 0.006**	1	5.1158	1.4355			*	*
			2	0.4178	0.3185				
			3	0.1840	0.0409	*			
			4	0.2223	0.1120	*			
E5	$\chi 2$ = 13.780	**p = 0.003**	1	3.4026	1.6217			***	
			2	0.3688	0.2497				
			3	0.1612	0.0549	***			
			4	0.3150	0.1939				

Supplementary Table 2. Power spectra of the theta band in the insulted and healthy hemispheres under normothermic and hypothermic conditions. The Kruskal-Wallis test was computed to perform the multiple comparisons within the 4 conditions: (1) the healthy hemisphere under normothermia (1), the insulted hemisphere under normothermia (2), the healthy hemisphere under hypothermia (3), and the insulted hemisphere under hypothermia (4). Dunn´s test was executed to determine which specific subanalyses revealed statistically significant differences. Both analyses were applied at 0h, 4h, 8h, 12h, and 16h. After Dunn´s test estimations, a Holm-Bonferroni adjustment was performed for multiple comparisons over time (p =0.05/6). Therefore, the adjusted p-value was set at 0.0083. The results from Dunn´s test shown in Figures 1-5 are the ones with p-values below 0.0083 in the general iteration of the Kruskal Wallis test (**p-values underlined and bold)**. In Dunn´s test, the statistically significant changes in the power spectrum of the frequency bands within conditions are represented as * when the p-value was equal to ≤0.05, ** when it was equal to ≤0.01, and as *** when it was equal to ≤0.001. KKW, Kruskal-Wallis test; M, mean; SD, standard deviation, $\chi 2$, chi-square.

ALPHA

Pre-MCAo

**E1**	KKW	$\chi2$ = 2.764	
		p = 0.430	
	**1**	M= 1.2530	SD= 0.4035
	**2**	M= 1.3096	SD= 0.4639
	**3**	M= 0.9808	SD= 0.1852
	**4**	M= 1.2895	SD= 0.6045
**E2**	KKW	$\chi2$ = 4.158	
		p = 0.245	
	**1**	M= 1.1128	SD= 0.3669
	**2**	M= 1.2880	SD= 0.3752
	**3**	M= 0.9973	SD= 0.1639
	**4**	M= 1.4142	SD= 0.4605
**E3**	KKW	$\chi2$ = 6.160	
		p = 0.104	
	**1**	M= 1.1232	SD= 0.2384
	**2**	M= 1.7850	SD= 0.8414
	**3**	M= 0.9470	SD= 0.1115
	**4**	M= 1.3507	SD= 0.3906
**E4**	KKW	$\chi2$ = 6.435	
		p = 0.092	
	**1**	M= 1.1532	SD= 0.2638
	**2**	M= 2.2130	SD= 0.9940
	**3**	M= 1.3262	SD= 0.8543
	**4**	M= 1.3803	SD= 0.3016
**E5**	KKW	$\chi2$ = 7.782	
		p = 0.051	
	**1**	M= 1.1926	SD= 0.5847
	**2**	M= 2.5980	SD= 1.0942
	**3**	M= 1.2093	SD= 0.4165
	**4**	M= 1.1950	SD= 0.4751

0h

**E1**	KKW	$\chi2$ = 2.202		Dunn´s test			
		p = 0.532		**1**	**2**	**3**	**4**
	**1**	M= 1.1462	SD= 0.4317				
	**2**	M= 1.1284	SD= 0.4390				
	**3**	M= 0.8618	SD= 0.1629				
	**4**	M= 1.0873	SD= 0.5318				
**E2**	KKW	$\chi2$ = 1.249		Dunn´s test			
		p = 0.741		**1**	**2**	**3**	**4**
	**1**	M= 1.0162	SD= 0.3492				
	**2**	M= 1.0894	SD= 0.5218				
	**3**	M= 0.9152	SD= 0.1737				
	**4**	M= 1.0750	SD= 0.4279				
**E3**	KKW	$\chi2$ = 3.115		Dunn´s test			
		p = 0.374		**1**	**2**	**3**	**4**
	**1**	M= 0.8548	SD= 0.5203				
	**2**	M= 1.5468	SD= 0.8695				
	**3**	M= 0.9690	SD= 0.0931				
	**4**	M= 1.0330	SD= 0.4059				
**E4**	KKW	$\chi2$ = 9.735		Dunn´s test			
		p = 0.021		**1**	**2**	**3**	**4**
	**1**	M= 1.0370	SD= 0.3500				
	**2**	M= 0.6960	SD= 0.3363				
	**3**	M= 0.9752	SD= 0.0948				*
	**4**	M= 0.5148	SD= 0.1604			*	
**E5**	KKW	$\chi2$ = 6.735		Dunn´s test			
		p = 0.081		**1**	**2**	**3**	**4**
	**1**	M= 0.8126	SD= 0.5044				
	**2**	M= 0.7778	SD= 0.3482				
	**3**	M= 0.9747	SD= 0.0759				
	**4**	M= 0.4888	SD= 0.1760				

4h

**E1**	KKW	$\chi2$ = 8.672		Dunn´s test			
		p = 0.034		**1**	**2**	**3**	**4**
	**1**	M= 0.8882	SD= 0.5560				
	**2**	M= 1.0842	SD= 0.4922				
	**3**	M= 0.2683	SD= 0.0971				
	**4**	M= 0.3962	SD= 0.3056				
**E2**	KKW	$\chi2$ = 10.728		Dunn´s test			
		p = 0.013		**1**	**2**	**3**	**4**
	**1**	M= 0.9206	SD= 0.4567		**		
	**2**	M= 0.1760	SD= 0.0602	**			
	**3**	M= 0.4212	SD= 0.0798				
	**4**	M= 0.4872	SD= 0.3551				
**E3**	KKW	$\chi2$ = 10.834		Dunn´s test			
		p = 0.013		**1**	**2**	**3**	**4**
	**1**	M= 0.9076	SD= 0.3647		**		
	**2**	M= 0.2578	SD= 0.0872	**			
	**3**	M= 0.4402	SD= 0.0728				
	**4**	M= 0.3735	SD= 0.2142				
**E4**	KKW	$\chi2$ = 10.877		Dunn´s test			
		p = 0.012		**1**	**2**	**3**	**4**
	**1**	M= 0.8546	SD= 0.4304		*		*
	**2**	M= 0.3040	SD= 0.2523	*			
	**3**	M= 0.4467	SD= 0.0476				
	**4**	M= 0.2900	SD= 0.1407	*			
**E5**	KKW	$\chi2$ = 13.587		Dunn´s test			
		**<u>p = 0.004</u>**		**1**	**2**	**3**	**4**
	**1**	M= 0.8834	SD= 0.3349		**		**
	**2**	M= 0.2420	SD= 0.0455	**			
	**3**	M= 0.3533	SD= 0.0862				
	**4**	M= 0.2400	SD= 0.1130	**			

8h

**E1**	KKW	$\chi2$ = 17.850		Dunn´s test			
		**<u>p = 0.000</u>**		**1**	**2**	**3**	**4**
	**1**	M= 1.1302	SD= 0.4188			**	
	**2**	M= 1.0352	SD= 0.4135			**	
	**3**	M= 0.0706	SD= 0.0263	**	**		
	**4**	M= 0.2548	SD= 0.1618				
**E2**	KKW	$\chi2$ = 18.002		Dunn´s test			
		**<u>p = 0.000</u>**		**1**	**2**	**3**	**4**
	**1**	M= 0.9656	SD= 0.4550			***	
	**2**	M= 0.1246	SD= 0.0208				
	**3**	M= 0.0788	SD= 0.0116	***			*
	**4**	M= 0.3118	SD= 0.1639			*	
**E3**	KKW	$\chi2$ = 16.147		Dunn´s test			
		**<u>p = 0.001</u>**		**1**	**2**	**3**	**4**
	**1**	M= 0.7894	SD= 0.4721			***	
	**2**	M= 0.2360	SD= 0.0581				
	**3**	M= 0.0900	SD= 0.0096	***			
	**4**	M= 0.2482	SD= 0.0786				
**E4**	KKW	$\chi2$ = 13.064		Dunn´s test			
		**<u>p = 0.005</u>**		**1**	**2**	**3**	**4**
	**1**	M= 0.8698	SD= 0.3225			*	**
	**2**	M= 0.2900	SD= 0.1219				
	**3**	M= 0.1957	SD= 0.0612	*			
	**4**	M= 0.1783	SD= 0.0523	**			
**E5**	KKW	$\chi2$ = 11.387		Dunn´s test			
		p = 0.010		**1**	**2**	**3**	**4**
	**1**	M= 0.9624	SD= 0.1875		*	*	
	**2**	M= 0.1966	SD= 0.0651	*			
	**3**	M= 0.2058	SD= 0.0497	*			
	**4**	M= 0.2217	SD= 0.0578				

12h

**E1**	KKW	$\chi2$ = 17.451		Dunn´s test			
		**<u>p = 0.001</u>**		**1**	**2**	**3**	**4**
	**1**	M= 1.0744	SD= 0.4348			***	
	**2**	M= 0.1778	SD= 0.1289				
	**3**	M= 0.0365	SD= 0.0204	***			
	**4**	M= 0.2497	SD= 0.1346				
**E2**	KKW	$\chi2$ = 18.023		Dunn´s test			
		**<u>p = 0.000</u>**		**1**	**2**	**3**	**4**
	**1**	M= 0.8754	SD= 0.5198			***	
	**2**	M= 0.1222	SD= 0.0354				
	**3**	M= 0.0332	SD= 0.0092	***			
	**4**	M= 0.2022	SD= 0.1163				
**E3**	KKW	$\chi2$ = 17.925		Dunn´s test			
		**<u>p = 0.000</u>**		**1**	**2**	**3**	**4**
	**1**	M= 0.8882	SD= 0.4762			***	
	**2**	M= 0.2042	SD= 0.0727				
	**3**	M= 0.0490	SD= 0.0102	***			
	**4**	M= 0.1750	SD= 0.0669				
**E4**	KKW	$\chi2$ = 14.881		Dunn´s test			
		**<u>p = 0.002</u>**		**1**	**2**	**3**	**4**
	**1**	M= 1.0524	SD= 0.3284			***	
	**2**	M= 0.2346	SD= 0.1730				
	**3**	M= 0.0893	SD= 0.0401	***			
	**4**	M= 0.1533	SD= 0.0635				
**E5**	KKW	$\chi2$ = 16.133		Dunn´s test			
		**<u>p = 0.001</u>**		**1**	**2**	**3**	**4**
	**1**	M= 0.9800	SD= 0.3131			**	*
	**2**	M= 0.1820	SD= 0.0581				
	**3**	M= 0.0782	SD= 0.0147	**			
	**4**	M= 0.0913	SD= 0.0590	*			

16h

**E1**	KKW	$\chi2$ = 18.443		Dunn´s test			
		**<u>p = 0.000</u>**		**1**	**2**	**3**	**4**
	**1**	M= 0.9878	SD= 0.4095			***	
	**2**	M= 0.1234	SD= 0.0708				
	**3**	M= 0.0151	SD= 0.0066	***			*
	**4**	M= 0.1995	SD= 0.0968			*	
**E2**	KKW	$\chi2$ = 19.002		Dunn´s test			
		**<u>p = 0.000</u>**		**1**	**2**	**3**	**4**
	**1**	M= 0.9630	SD= 0.4893		*	***	
	**2**	M= 0.0786	SD= 0.0413	*			
	**3**	M= 0.0193	SD= 0.0103	***			*
	**4**	M= 0.2052	SD= 0.0757			*	
**E3**	KKW	$\chi2$ = 17.769		Dunn´s test			
		**<u>p = 0.000</u>**		**1**	**2**	**3**	**4**
	**1**	M= 0.8310	SD= 0.4940			***	
	**2**	M= 0.1598	SD= 0.0518				
	**3**	M= 0.0215	SD= 0.0129	***			
	**4**	M= 0.1627	SD= 0.0424				
**E4**	KKW	$\chi2$ = 17.344		Dunn´s test			
		**<u>p = 0.001</u>**		**1**	**2**	**3**	**4**
	**1**	M= 1.0362	SD= 0.3550			***	*
	**2**	M= 0.2240	SD= 0.0770				
	**3**	M= 0.0509	SD= 0.0386	***			
	**4**	M= 0.1250	SD= 0.0644	*			
**E5**	KKW	$\chi2$ = 15.032		Dunn´s test			
		**<u>p = 0.002</u>**		**1**	**2**	**3**	**4**
	**1**	M= 0.9012	SD= 0.3203			***	*
	**2**	M= 0.1334	SD= 0.0619				
	**3**	M= 0.0485	SD= 0.0101	***			
	**4**	M= 0.0863	SD= 0.0733	*			

Supplementary Table 3. Power spectra of the alpha band in the insulted and healthy hemispheres under normothermic and hypothermic conditions. The Kruskal-Wallis test was computed to perform the multiple comparisons within the 4 conditions: (1) the healthy hemisphere under normothermia (1), the insulted hemisphere under normothermia (2), the healthy hemisphere under hypothermia (3), and the insulted hemisphere under hypothermia (4). Dunn´s test was executed to determine which specific subanalyses revealed statistically significant differences. Both analyses were applied at 0h, 4h, 8h, 12h, and 16h. After Dunn´s test estimations, a Holm-Bonferroni adjustment was performed for multiple comparisons over time ($p = 0.05/6$). Therefore, the adjusted p-value was set at 0.0083. The results from Dunn´s test shown in Figures 1-5 are the ones with p-values below 0.0083 in the general iteration of the Kruskal Wallis test (**<u>p-values underlined and bold)</u>**. In Dunn´s test, the statistically significant changes in the power spectrum of the frequency bands within conditions are represented as * when the p-value was equal to ≤ 0.05, ** when it was equal to ≤ 0.01, and as *** when it was equal to ≤ 0.001. KKW, Kruskal-Wallis test; M, mean; SD, standard deviation, $\chi2$, chi-square.

BETA

Pre-MCAo

		M	SD
E1	KKW	χ2 = 1.010	p = 0.799
	1	0.3328	0.1614
	2	0.3808	0.1689
	3	0.3627	0.1061
	4	0.4318	0.1554
E2	KKW	χ2 = 2.034	p = 0.565
	1	0.4198	0.1768
	2	0.3218	0.2190
	3	0.4400	0.1099
	4	0.4337	0.1260
E3	KKW	χ2 = 0.272	p = 0.965
	1	0.3794	0.1723
	2	0.3638	0.2125
	3	0.4128	0.0887
	4	0.3937	0.1931
E4	KKW	χ2 = 2.912	p = 0.405
	1	0.3628	0.2073
	2	0.5822	0.2619
	3	0.4653	0.0965
	4	0.4253	0.1369
E5	KKW	χ2 = 3.447	p = 0.328
	1	0.3254	0.1425
	2	0.4152	0.2242
	3	0.4458	0.0676
	4	0.3783	0.1332

0h

		M	SD	Dunn´s test 1	2	3	4
E1	KKW	χ2 = 2.107	p = 0.551				
	1	0.2942	0.2157				
	2	0.3662	0.1215				
	3	0.3777	0.0990				
	4	0.3548	0.1094				
E2	KKW	χ2 = 12.892	**p = 0.005**				
	1	0.3766	0.1797				
	2	0.1104	0.0208			**	
	3	0.4713	0.0854		**		
	4	0.3510	0.0912				
E3	KKW	χ2 = 13.864	**p = 0.003**				
	1	0.3648	0.2005				
	2	0.1088	0.0370			**	
	3	0.4237	0.0703		**		
	4	0.1617	0.0635				
E4	KKW	χ2 = 12.560	**p = 0.006**				
	1	0.3434	0.1333				
	2	0.3106	0.0228			*	
	3	0.4917	0.0816		*		**
	4	0.2697	0.0724			**	
E5	KKW	χ2 = 13.030	**p = 0.005**				
	1	0.3534	0.1752				
	2	0.1330	0.0853			*	
	3	0.4613	0.0474		*		*
	4	0.1325	0.0570			*	

4h

		M	SD	Dunn´s test 1	2	3	4
E1	KKW	χ2 = 13.119	**p = 0.004**				
	1	0.3326	0.1790				*
	2	0.3300	0.1201				**
	3	0.2467	0.0493				
	4	0.0813	0.0366	*	**		
E2	KKW	χ2 = 14.289	**p = 0.003**				
	1	0.4290	0.1235				**
	2	0.3476	0.1581				
	3	0.2923	0.0453				
	4	0.0978	0.0331	**			
E3	KKW	χ2 = 15.191	**p = 0.002**				
	1	0.3204	0.1580		*		
	2	0.0538	0.0364	*		**	
	3	0.2692	0.0324		**		
	4	0.1180	0.0517				
E4	KKW	χ2 = 15.779	**p = 0.001**				
	1	0.3932	0.1724				*
	2	0.5654	0.1854				***
	3	0.2668	0.0637				
	4	0.1030	0.0517	*	***		
E5	KKW	χ2 = 16.427	**p = 0.001**				
	1	0.2752	0.2312		*		
	2	0.0410	0.0156	*		**	
	3	0.2168	0.0300		**		*
	4	0.0593	0.0250			*	

8h

		M	SD	Dunn´s test 1	2	3	4
E1	KKW	χ2 = 17.214	**p = 0.001**				
	1	0.3300	0.1663				**
	2	0.2990	0.1088				**
	3	0.0850	0.0296				
	4	0.0432	0.0251	**	**		
E2	KKW	χ2 = 17.758	**p = 0.000**				
	1	0.3548	0.1560				***
	2	0.1154	0.0259				
	3	0.1133	0.0203				
	4	0.0393	0.0090	***			
E3	KKW	χ2 = 17.748	**p = 0.000**				
	1	0.3914	0.1720		**		**
	2	0.0444	0.0268	**			
	3	0.1268	0.0273				
	4	0.0455	0.0205	**			
E4	KKW	χ2 = 18.406	**p = 0.000**				
	1	0.3338	0.1351				***
	2	0.2946	0.0161				**
	3	0.0958	0.0193				
	4	0.0378	0.0079	***	**		
E5	KKW	χ2 = 17.035	**p = 0.001**				
	1	0.3768	0.1544		**		**
	2	0.0350	0.0219	**			
	3	0.0843	0.0197				
	4	0.0267	0.0082	**			

12h

		M	SD	Dunn´s test 1	2	3	4
E1	KKW	χ2 = 14.643	**p = 0.002**				
	1	0.3152	0.1829			**	**
	2	0.0698	0.0181				
	3	0.0440	0.0126	**			
	4	0.0422	0.0194	**			
E2	KKW	χ2 = 18.296	**p = 0.000**				
	1	0.3718	0.1624				***
	2	0.0992	0.0234				
	3	0.0778	0.0149				
	4	0.0207	0.0059	***			
E3	KKW	χ2 = 17.040	**p = 0.001**				
	1	0.4114	0.1460		**		**
	2	0.0190	0.0048	**			
	3	0.0562	0.0140				
	4	0.0242	0.0149	**			
E4	KKW	χ2 = 16.649	**p = 0.001**				
	1	0.3346	0.1282				**
	2	0.2988	0.0170				*
	3	0.0380	0.0060				
	4	0.0283	0.0110	**	*		
E5	KKW	χ2 = 15.719	**p = 0.001**				
	1	0.3570	0.1505		*		***
	2	0.0300	0.0216	*			
	3	0.0350	0.0084				
	4	0.0185	0.0042	***			

16h

		M	SD	Dunn´s test 1	2	3	4
E1	KKW	χ2 = 16.119	**p = 0.001**				
	1	0.3028	0.1722			***	
	2	0.0442	0.0211				
	3	0.0153	0.0066	***			
	4	0.0402	0.0262				
E2	KKW	χ2 = 18.614	**p = 0.000**				
	1	0.4500	0.1409				***
	2	0.2942	0.1901				**
	3	0.0357	0.0066				
	4	0.0137	0.0048	***	**		
E3	KKW	χ2 = 12.392	**p = 0.006**				
	1	0.3904	0.1966		*	**	
	2	0.0150	0.0055	*			
	3	0.0157	0.0092	**			
	4	0.0197	0.0054				
E4	KKW	χ2 = 16.338	**p = 0.001**				
	1	0.3510	0.1560			*	
	2	0.4830	0.1237			**	*
	3	0.0103	0.0037	*	**		
	4	0.0135	0.0059		*		
E5	KKW	χ2 = 15.304	**p = 0.002**				
	1	0.3420	0.1629				**
	2	0.0340	0.0147				**
	3	0.0127	0.0073				
	4	0.0123	0.0050	**	**		

Supplementary Table 4. Power spectra of the beta band in the insulted and healthy hemispheres under normothermic and hypothermic conditions. The Kruskal-Wallis test was computed to perform the multiple comparisons within the 4 conditions: (1) the healthy hemisphere under normothermia (1), the insulted hemisphere under normothermia (2), the healthy hemisphere under hypothermia (3), and the insulted hemisphere under hypothermia (4). Dunn´s test was executed to determine which specific subanalyses revealed statistically significant differences. Both analyses were applied at 0h, 4h, 8h, 12h, and 16h. After Dunn´s test estimations, a Holm-Bonferroni adjustment was performed for multiple comparisons over time (p =0.05/6). Therefore, the adjusted p-value was set at 0.0083. The results from Dunn´s test shown in Figures 1-5 are the ones with p-values below 0.0083 in the general iteration of the Kruskal Wallis test (**p-values underlined and bold)**. In Dunn´s test, the statistically significant changes in the power spectrum of the frequency bands within conditions are represented as * when the p-value was equal to ≤0.05, ** when it was equal to ≤0.01, and as *** when it was equal to ≤0.001. KKW, Kruskal-Wallis test; M, mean; SD, standard deviation, χ2, chi-square.

GAMMA

Pre-MCAo

Exp.		M	SD
E1	KKW	χ2 = 1.446	p = 0.695
E1	1	M= 0.0358	SD= 0.0178
E1	2	M= 0.0392	SD= 0.0265
E1	3	M= 0.0460	SD= 0.0058
E1	4	M= 0.0447	SD= 0.0130
E2	KKW	χ2 = 1.485	p = 0.686
E2	1	M= 0.0364	SD= 0.0145
E2	2	M= 0.0438	SD= 0.0235
E2	3	M= 0.0455	SD= 0.0058
E2	4	M= 0.0420	SD= 0.0139
E3	KKW	χ2 = 1.680	p = 0.641
E3	1	M= 0.0374	SD= 0.0169
E3	2	M= 0.0466	SD= 0.0206
E3	3	M= 0.0468	SD= 0.0089
E3	4	M= 0.0437	SD= 0.0160
E4	KKW	χ2 = 3.272	p = 0.352
E4	1	M= 0.0368	SD= 0.0158
E4	2	M= 0.0494	SD= 0.0257
E4	3	M= 0.0512	SD= 0.0079
E4	4	M= 0.0462	SD= 0.0126
E5	KKW	χ2 = 4.568	p = 0.206
E5	1	M= 0.0346	SD= 0.0170
E5	2	M= 0.0548	SD= 0.0168
E5	3	M= 0.0522	SD= 0.0061
E5	4	M= 0.0433	SD= 0.0160

0h

Exp.		M	SD	Dunn´s test 1	2	3	4
E1	KKW	χ2 = 3.231	p = 0.357	1	2	3	4
E1	1	M= 0.0370	SD= 0.0148				
E1	2	M= 0.0404	SD= 0.0186				
E1	3	M= 0.0467	SD= 0.0070				
E1	4	M= 0.0343	SD= 0.0171				
E2	KKW	χ2 = 3.453	p = 0.327	1	2	3	4
E2	1	M= 0.0390	SD= 0.0175				
E2	2	M= 0.0446	SD= 0.0146				
E2	3	M= 0.0437	SD= 0.0090				
E2	4	M= 0.0347	SD= 0.0119				
E3	KKW	χ2 = 13.274	**p = 0.004**	1	2	3	4
E3	1	M= 0.0360	SD= 0.0168				
E3	2	M= 0.0166	SD= 0.0090			**	
E3	3	M= 0.0493	SD= 0.0073		**		
E3	4	M= 0.0248	SD= 0.0075				
E4	KKW	χ2 = 9.805	p = 0.020	1	2	3	4
E4	1	M= 0.0342	SD= 0.0182				
E4	2	M= 0.0232	SD= 0.0140			*	
E4	3	M= 0.0513	SD= 0.0069		*		*
E4	4	M= 0.0233	SD= 0.0099			*	
E5	KKW	χ2 = 11.354	p = 0.010	1	2	3	4
E5	1	M= 0.0372	SD= 0.0181				
E5	2	M= 0.0258	SD= 0.0107				
E5	3	M= 0.0505	SD= 0.0059				*
E5	4	M= 0.0200	SD= 0.0073			*	

4h

Exp.		M	SD	Dunn´s test 1	2	3	4
E1	KKW	χ2 = 9.503	p = 0.023	1	2	3	4
E1	1	M= 0.0342	SD= 0.0184				
E1	2	M= 0.0382	SD= 0.0181				
E1	3	M= 0.0157	SD= 0.0031				
E1	4	M= 0.0187	SD= 0.0077				
E2	KKW	χ2 = 10.997	p = 0.012	1	2	3	4
E2	1	M= 0.0360	SD= 0.0187				
E2	2	M= 0.0428	SD= 0.0168				*
E2	3	M= 0.0168	SD= 0.0046				
E2	4	M= 0.0160	SD= 0.0097		*		
E3	KKW	χ2 = 14.108	**p = 0.003**	1	2	3	4
E3	1	M= 0.0376	SD= 0.0184		**		
E3	2	M= 0.0078	SD= 0.0016	**			
E3	3	M= 0.0168	SD= 0.0035				
E3	4	M= 0.0137	SD= 0.0062				
E4	KKW	χ2 = 11.650	p = 0.009	1	2	3	4
E4	1	M= 0.0370	SD= 0.0176		**		
E4	2	M= 0.0094	SD= 0.0038	**			
E4	3	M= 0.0180	SD= 0.0050				
E4	4	M= 0.0162	SD= 0.0082				
E5	KKW	χ2 = 14.514	**p = 0.002**	1	2	3	4
E5	1	M= 0.0380	SD= 0.0114		**		*
E5	2	M= 0.0102	SD= 0.0028	**			
E5	3	M= 0.0172	SD= 0.0044				
E5	4	M= 0.0147	SD= 0.0061	*			

8h

Exp.		M	SD	Dunn´s test 1	2	3	4
E1	KKW	χ2 = 14.537	**p = 0.002**	1	2	3	4
E1	1	M= 0.0310	SD= 0.0184				
E1	2	M= 0.0364	SD= 0.0177			*	*
E1	3	M= 0.0082	SD= 0.0028		*		
E1	4	M= 0.0083	SD= 0.0021		*		
E2	KKW	χ2 = 15.898	**p = 0.001**	1	2	3	4
E2	1	M= 0.0346	SD= 0.0167				*
E2	2	M= 0.0404	SD= 0.0167			*	*
E2	3	M= 0.0098	SD= 0.0018		*		
E2	4	M= 0.0088	SD= 0.0023	*	*		
E3	KKW	χ2 = 15.197	**p = 0.002**	1	2	3	4
E3	1	M= 0.0352	SD= 0.0158		***		*
E3	2	M= 0.0064	SD= 0.0015	***			
E3	3	M= 0.0096	SD= 0.0020				
E3	4	M= 0.0083	SD= 0.0012	*			
E4	KKW	χ2 = 14.103	**p = 0.003**	1	2	3	4
E4	1	M= 0.0296	SD= 0.0166		***		
E4	2	M= 0.0060	SD= 0.0023	***			
E4	3	M= 0.0107	SD= 0.0039				
E4	4	M= 0.0103	SD= 0.0042				
E5	KKW	χ2 = 12.620	**p = 0.006**	1	2	3	4
E5	1	M= 0.0340	SD= 0.0185		**		
E5	2	M= 0.0066	SD= 0.0021	**			
E5	3	M= 0.0098	SD= 0.0036				
E5	4	M= 0.0107	SD= 0.0029				

12h

Exp.		M	SD	Dunn´s test 1	2	3	4
E1	KKW	χ2 = 16.342	**p = 0.001**	1	2	3	4
E1	1	M= 0.0354	SD= 0.0192			**	
E1	2	M= 0.0328	SD= 0.0169			*	
E1	3	M= 0.0042	SD= 0.0018	**	*		
E1	4	M= 0.0058	SD= 0.0017				
E2	KKW	χ2 = 16.687	**p = 0.001**	1	2	3	4
E2	1	M= 0.0358	SD= 0.0184			**	
E2	2	M= 0.0392	SD= 0.0167			**	
E2	3	M= 0.0041	SD= 0.0014	**	**		
E2	4	M= 0.0063	SD= 0.0022				
E3	KKW	χ2 = 12.054	**p = 0.007**	1	2	3	4
E3	1	M= 0.0330	SD= 0.0169			**	*
E3	2	M= 0.0054	SD= 0.0018				
E3	3	M= 0.0041	SD= 0.0010	**			
E3	4	M= 0.0047	SD= 0.0019	*			
E4	KKW	χ2 = 11.571	p = 0.009	1	2	3	4
E4	1	M= 0.0414	SD= 0.0146		*	*	
E4	2	M= 0.0044	SD= 0.0028	*			
E4	3	M= 0.0051	SD= 0.0020	*			
E4	4	M= 0.0055	SD= 0.0024				
E5	KKW	χ2 = 15.406	**p = 0.002**	1	2	3	4
E5	1	M= 0.0420	SD= 0.0163		*	***	
E5	2	M= 0.0054	SD= 0.0015	*			
E5	3	M= 0.0040	SD= 0.0011	***			
E5	4	M= 0.0068	SD= 0.0019				

16h

Exp.		M	SD	Dunn´s test 1	2	3	4
E1	KKW	χ2 = 16.728	**p = 0.001**	1	2	3	4
E1	1	M= 0.0336	SD= 0.0148			**	**
E1	2	M= 0.0142	SD= 0.0049				
E1	3	M= 0.0013	SD= 0.0009	**			
E1	4	M= 0.0013	SD= 0.0006	**			
E2	KKW	χ2 = 17.494	**p = 0.001**	1	2	3	4
E2	1	M= 0.0360	SD= 0.0177			***	*
E2	2	M= 0.0152	SD= 0.0069			*	
E2	3	M= 0.0012	SD= 0.0007	***	*		
E2	4	M= 0.0030	SD= 0.0021	*			
E3	KKW	χ2 = 14.631	**p = 0.002**	1	2	3	4
E3	1	M= 0.0390	SD= 0.0160			***	
E3	2	M= 0.0024	SD= 0.0013				
E3	3	M= 0.0010	SD= 0.0005	***			
E3	4	M= 0.0020	SD= 0.0009				
E4	KKW	χ2 = 13.811	**p = 0.003**	1	2	3	4
E4	1	M= 0.0370	SD= 0.0180			***	
E4	2	M= 0.0023	SD= 0.0010				
E4	3	M= 0.0013	SD= 0.0005	***			
E4	4	M= 0.0022	SD= 0.0012				
E5	KKW	χ2 = 16.548	**p = 0.001**	1	2	3	4
E5	1	M= 0.0390	SD= 0.0183			***	
E5	2	M= 0.0032	SD= 0.0015				
E5	3	M= 0.0008	SD= 0.0005	***			
E5	4	M= 0.0025	SD= 0.0010				

Supplementary Table 5. Power spectra of the gamma band in the insulted and healthy hemispheres under normothermic and hypothermic conditions. The Kruskal-Wallis test was computed to perform the multiple comparisons within the 4 conditions: (1) the healthy hemisphere under normothermia (1), the insulted hemisphere under normothermia (2), the healthy hemisphere under hypothermia (3), and the insulted hemisphere under hypothermia (4). Dunn´s test was executed to determine which specific subanalyses revealed statistically significant differences. Both analyses were applied at 0h, 4h, 8h, 12h, and 16h. After Dunn´s test estimations, a Holm-Bonferroni adjustment was performed for multiple comparisons over time (p =0.05/6). Therefore, the adjusted p-value was set at 0.0083. The results from Dunn´s test shown in Figures 1-5 are the ones with p-values below 0.0083 in the general iteration of the Kruskal Wallis test (**p-values underlined and bold)**. In Dunn´s test, the statistically significant changes in the power spectrum of the frequency bands within conditions are represented as * when the p-value was equal to ≤0.05, ** when it was equal to ≤0.01, and as *** when it was equal to ≤0.001. KKW, Kruskal-Wallis test; M, mean; SD, standard deviation, χ2, chi-square.

DELTA

	PreSD		SD		postSD	
	Control	Hypothermia	Control	Hypothermia	Control	Hypothermia
**E1L**	M = 2.2910 SD= 2.6386	M = 4.5858 SD= 4.2389	M = 0.2672 SD= 0.3707	M = 0.4320 SD= 0.3001	M = 1.6848 SD= 1.2382489	M = 3.8132 SD= 2.9281
	U = 230		U = 169		U = 209	
	p = 0.02		**p = 0.001**		**p = 0.007**	
**E2L**	M = 0.8696 SD= 0.6296	M = 1.9228 SD= 1.4419	M = 0.1821 SD= 0.0541	M = 0.1974 SD= 0.1141	M = 0.4562 SD= .3222130	M = 0.9394 SD= 0.7414
	U = 31		U = 95		U = 67	
	**p = 0.001**		p = 0.91		p = 0.164	
**E3L**	M = 1.7206 SD= 0.4081	M = 0.7492 SD= 1.0494	M = 0.3516 SD= 0.1222	M = 0.1047 SD= 0.1795	M = 1.3344 SD= .3819558	M = 0.6084 SD= 1.1678
	U = 14		U = 13		U = 13	
	**p = 0.001**		**p = 0.001**		**p = 0.001**	

THETA

	PreSD		SD		postSD	
	Control	Hypothermia	Control	Hypothermia	Control	Hypothermia
**E1L**	M = 1.0789 SD= 1.1869	M = 1.9386 SD= 1.9450	M = 0.0823 SD= 0.1068	M = 0.1406 SD= 0.1060	M = 0.8966 SD= 0.8090	M = 1.4576 SD= 1.3494
	U = 271		U = 232		U = 288	
	p = 0.106		p = 0.022		p = 0.186	
**E2L**	M = 0.2571 SD= 0.2343	M = 0.6684 SD= 0.8943	M = 0.0241 SD= 0.0115	M = 0.0341 SD= 0.0377	M = 0.1037 SD= 0.0648	M = 0.2506 SD= 0.3303
	U = 65		U = 89		U = 85	
	p = 0.137		p = 0.701		p = 0.571	
**E3L**	M = 0.4005 SD= 0.1071	M = 0.1527 SD= 0.3228	M = 0.0661 SD= 0.0274	M = 0.0152 SD= 0.0430	M = 0.3064 SD= 0.0519	M = 0.0943 SD= 0.2080
	U = 13		U = 13		U = 13	
	**p = 0.001**		**p = 0.001**		**p = 0.001**	

ALPHA

	PreSD		SD		postSD	
	Control	Hypothermia	Control	Hypothermia	Control	Hypothermia
**E1L**	M = 0.2845 SD= 0.3088	M = 0.3304 SD= 0.3211	M = 0.0204 SD= 0.0251	M = 0.0290 SD= 0.0227	M = 0.2099 SD= 0.1963	M = 0.2812 SD= 0.2094
	U = 321		U = 263.5		U = 286	
	p = 0.452		p = 0.081		p = 0.174	
**E2L**	M = 0.0703 SD= 0.0509	M = 0.1541 SD= 0.2348	M = 0.0072 SD= 0.0038	M = 0.0077 SD= 0.0073	M = 0.0323 SD= 0.0119	M = 0.0625 SD= 0.0848
	U = 83		U = 73		U = 92.5	
	p = 0.511		p = 0.265		p = 0.804	
**E3L**	M = 0.1054 SD= 0.0397	M = 0.0389 SD= 0.0792	M = 0.0159 SD= 0.0066	M = 0.0063 SD= 0.0167	M = 0.0788 SD= 0.0170	M = 0.0257 SD= 0.0471
	U = 13		U = 13		U = 14	
	**p = 0.001**		**p = 0.001**		**p = 0.001**	

BETHA						
	PreSD		SD		postSD	
	Control	Hypothermia	Control	Hypothermia	Control	Hypothermia
E1L	M = 0.0657 SD= 0.0510	M = 0.1554 SD= 0.1130	M = 0.0082 SD= 0.0061	M = 0.0236 SD= 0.0221	M = 0.0470 SD= 0.0179	M = 0.1254 SD= 0.0797
	U = 174		U = 196.5		U = 175.5	
	**p = 0.001**		**p = 0.004**		**p = 0.001**	
E2L	M = 0.0220 SD= 0.0056	M = 0.1044 SD= 0.0957	M = 0.0036 SD= 0.0012	M = 0.0167 SD= 0.0164	M = 0.0126 SD= 0.0033	M = 0.0708 SD= 0.0645
	U = 39		U = 61		U = 23	
	**p = 0.006**		p = 0.094		**p = 0.001**	
E3L	M = 0.0219 SD= 0.0052	M = 0.0426 SD= 0.0659	M = 0.0038 SD= 0.0011	M = 0.0143 SD= 0.0373	M = 0.0153 SD= 0.0042	M = 0.0241 SD= 0.0360
	U = 42.5		U = 48.5		U = 38.5	
	p = 0.029		p = 0.064		**p = 0.016**	

GAMMA						
	PreSD		SD		postSD	
	Control	Hypothermia	Control	Hypothermia	Control	Hypothermia
E1L	M = 0.0135 SD= 0.0070	M = 0.0159 SD= 0.0062	M = 0.0021 SD= 0.0011	M = 0.0030 SD= 0.0015	M = 0.0120 SD= 0.0046	M = 0.0140 SD= 0.0056
	U = 260		U = 211		U = 297.5	
	p = 0.071		**p = 0.008**		p = 0.246	
E2L	M = 0.0095 SD= 0.0018	M = 0.0114 SD= 0.0041	M = 0.0017 SD= 0.0005	M = 0.0017 SD= 0.0006	M = 0.0046 SD= 0.0018	M = 0.0051 SD= 0.0036
	U = 71.5		U = 90.5		U = 86.5	
	p = 0.227		p = 0.734		p = 0.603	
E3L	M = 0.0086 SD= 0.0014	M = 0.0097 SD= 0.0038	M = 0.0016 SD= 0.0004	M = 0.0030 SD= 0.0060	M = 0.0034 SD= 0.0015	M = 0.0040 SD= 0.0032
	U = 73		U = 67		U = 83	
	p = 0.579		p = 0.39		p = 0.96	

Supplementary Table 6. Power spectra of frequency bands before, during, and after the SD formation in the insulted hemisphere under normothermic and hypothermic conditions. The U-Mann Whitney test was performed to discern the differences in the power spectrum of the five frequency bands between normothermia and hypothermia during the SD formation in E1L to E3L. After the U-Mann Whitney test, the Holm-Bonferroni adjustment was performed for multiple comparisons over time (p =0.05/3). Therefore, the adjusted p-value was set at 0.017. The results shown in Figure 6 are the ones with p-values below 0.0017 in the general iteration of the U-Mann Whitney test (**p-values underlined and bold)**. M, mean; SD, standard deviation, U, Mann-Whitney U test.

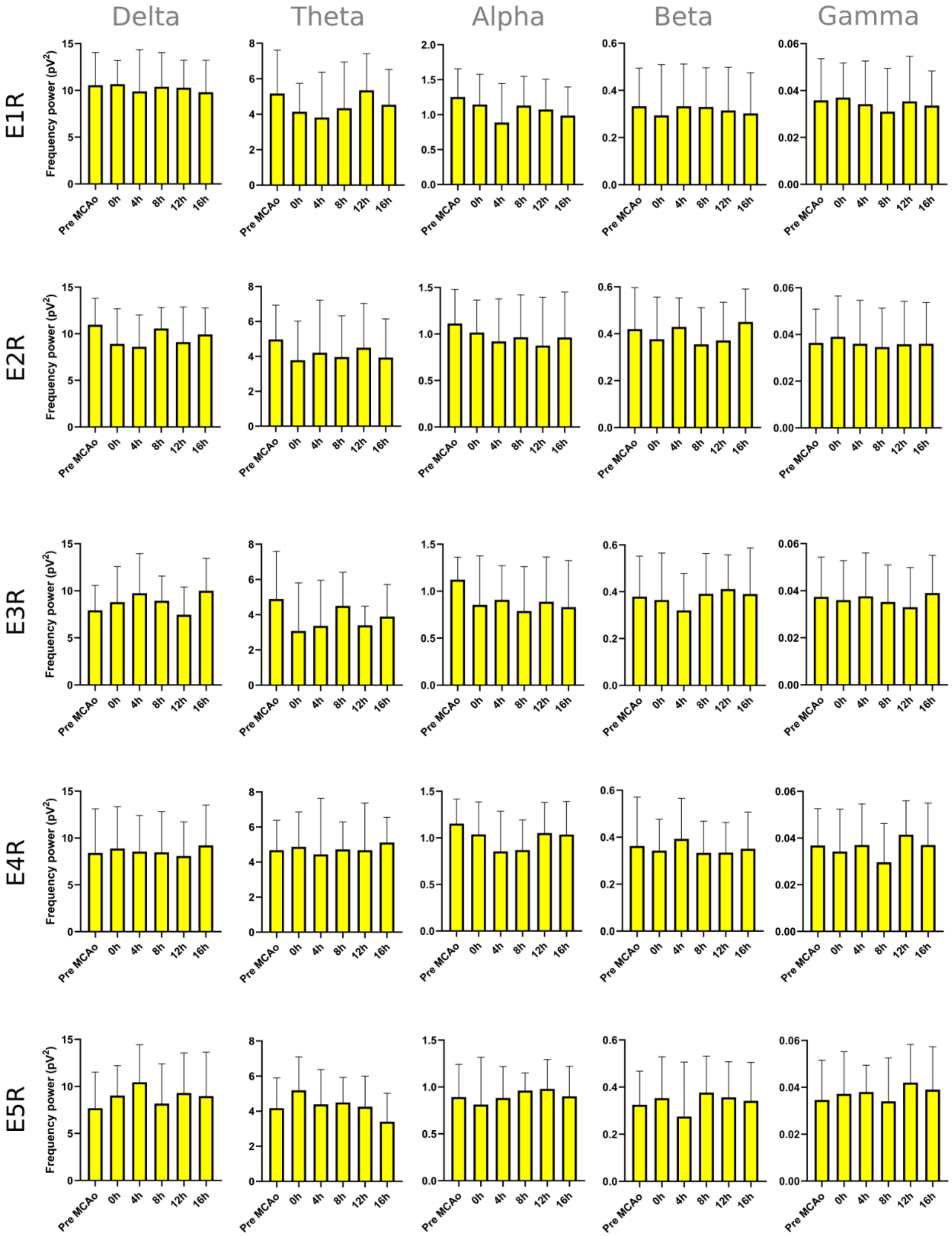

Supplementary Figure 1. Frequency bands in the right electrodes after MCAo in the control group over time: Healthy normothermic hemisphere. The 5-min signal segment before MCAo was used as the baseline to compare the changes in frequency bands at 0 h (5 min immediately after), 4 h, 8 h, 12 h, and 16 h after MCAo applying the Wilcoxon matched-pairs signed-rank test. The significant drop in the power spectrum of the frequencies is represented as * (≤ 0.05). MCAo, middle cerebral artery occlusion; pV^2, squared picovolts.

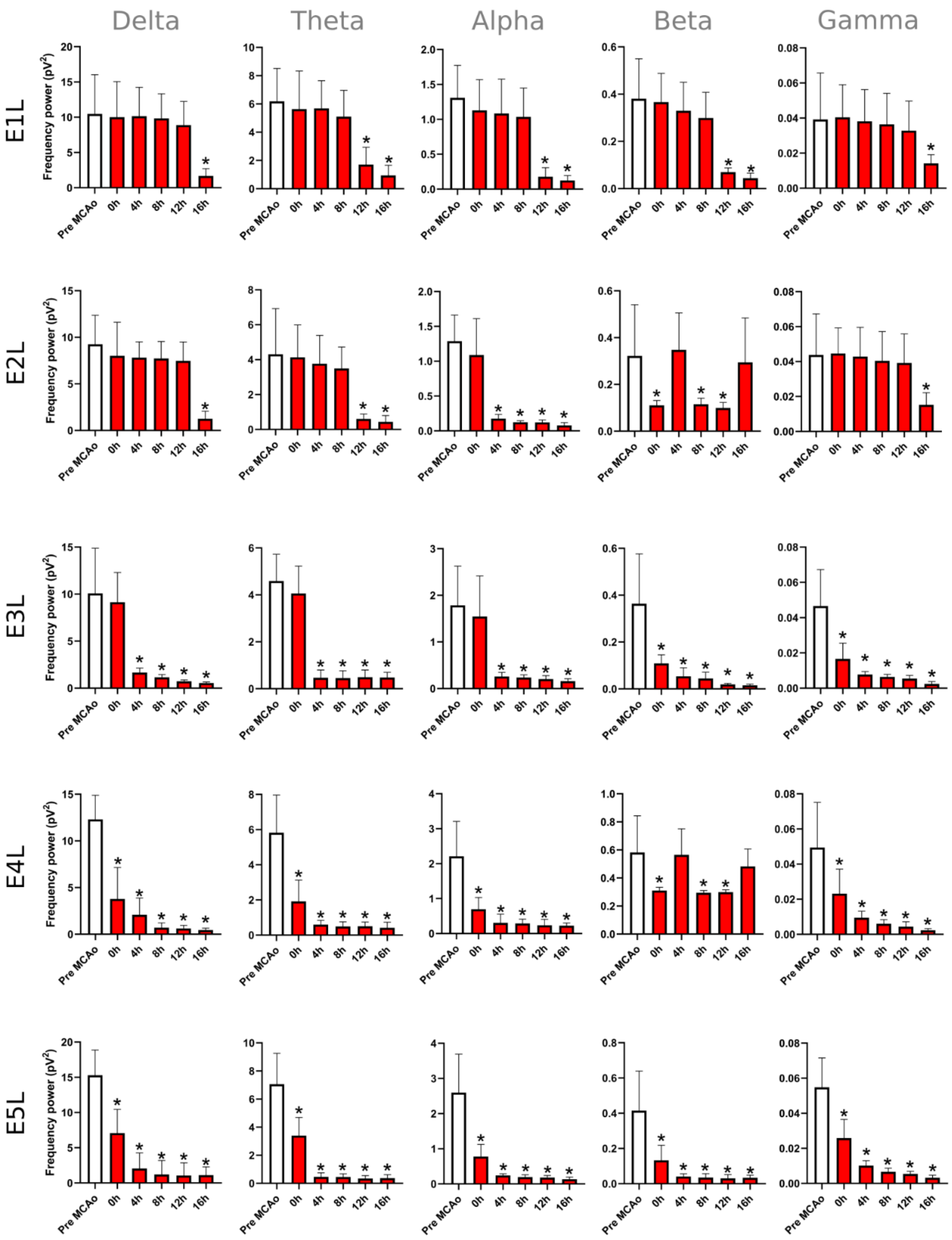

Supplementary Figure 2. Frequency bands in the left electrodes after MCAo in the control group over time: Insulted normothermic hemisphere. The 5-min signal segment before MCAo was used as the baseline to compare the changes in frequency bands at 0 h (5 min immediately after), 4 h, 8 h, 12 h, and 16 h after MCAo applying the Wilcoxon matched-pairs signed-rank test. The significant drop in the power spectrum of the frequencies is represented as * (≤ 0.05). MCAo, middle cerebral artery occlusion; pV^2, squared picovolts.

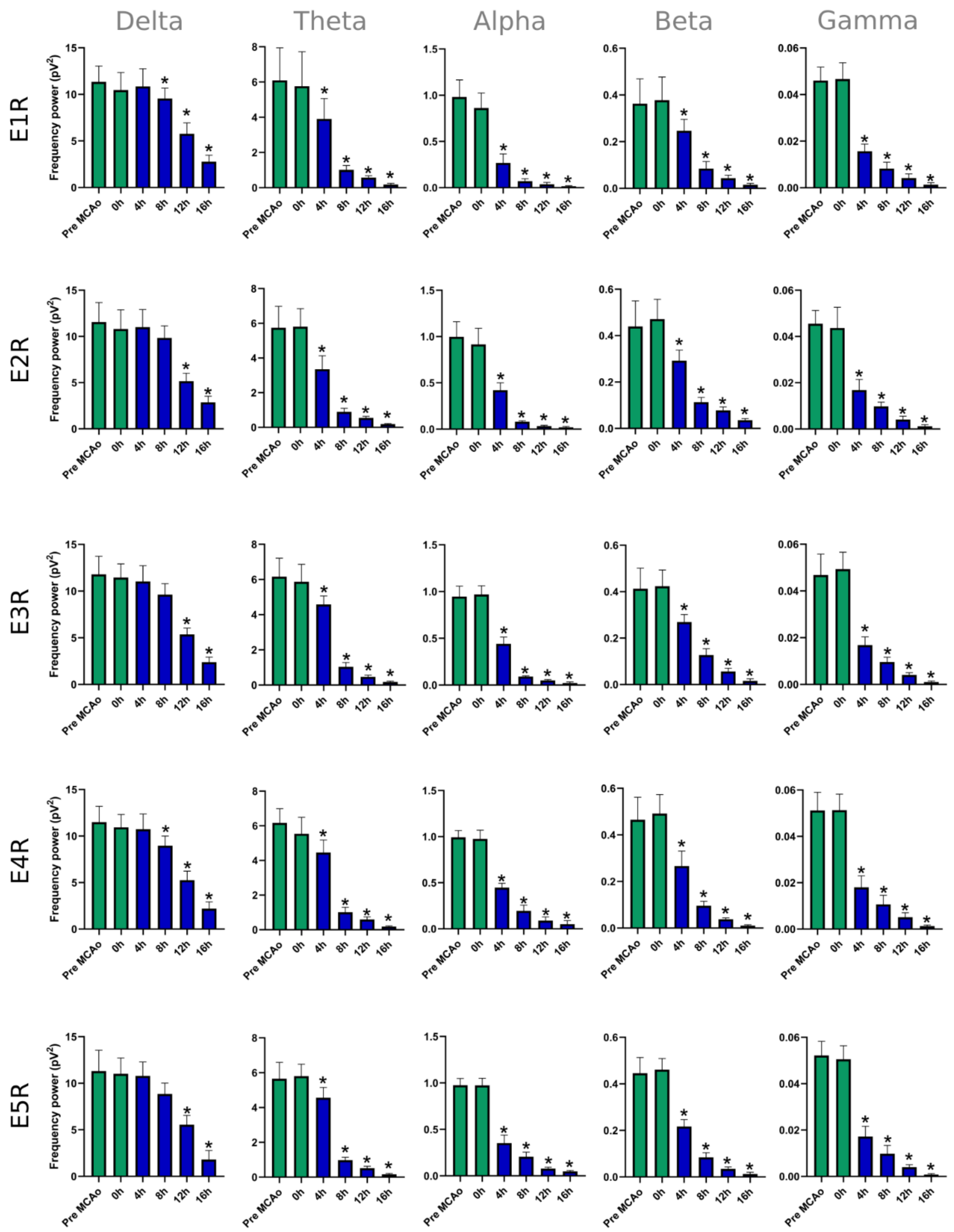

Supplementary Figure 3. Frequency bands in the right electrodes after MCAo in the hypothermia group over time: Healthy hypothermic hemisphere. Mild hypothermia was introduced since the first hour of MCAo and completely established at the fourth hour at 32°C. The 5-min signal segment before MCAo was used as the baseline to compare the changes in frequency bands at 0 h (5 min immediately after), 4 h, 8 h, 12 h, and 16 h after MCAo applying the Wilcoxon matched-pairs signed-rank test. The significant drop in the power spectrum of the frequencies is represented as * (≤ 0.05). MCAo, middle cerebral artery occlusion; pV^2, squared picovolts.

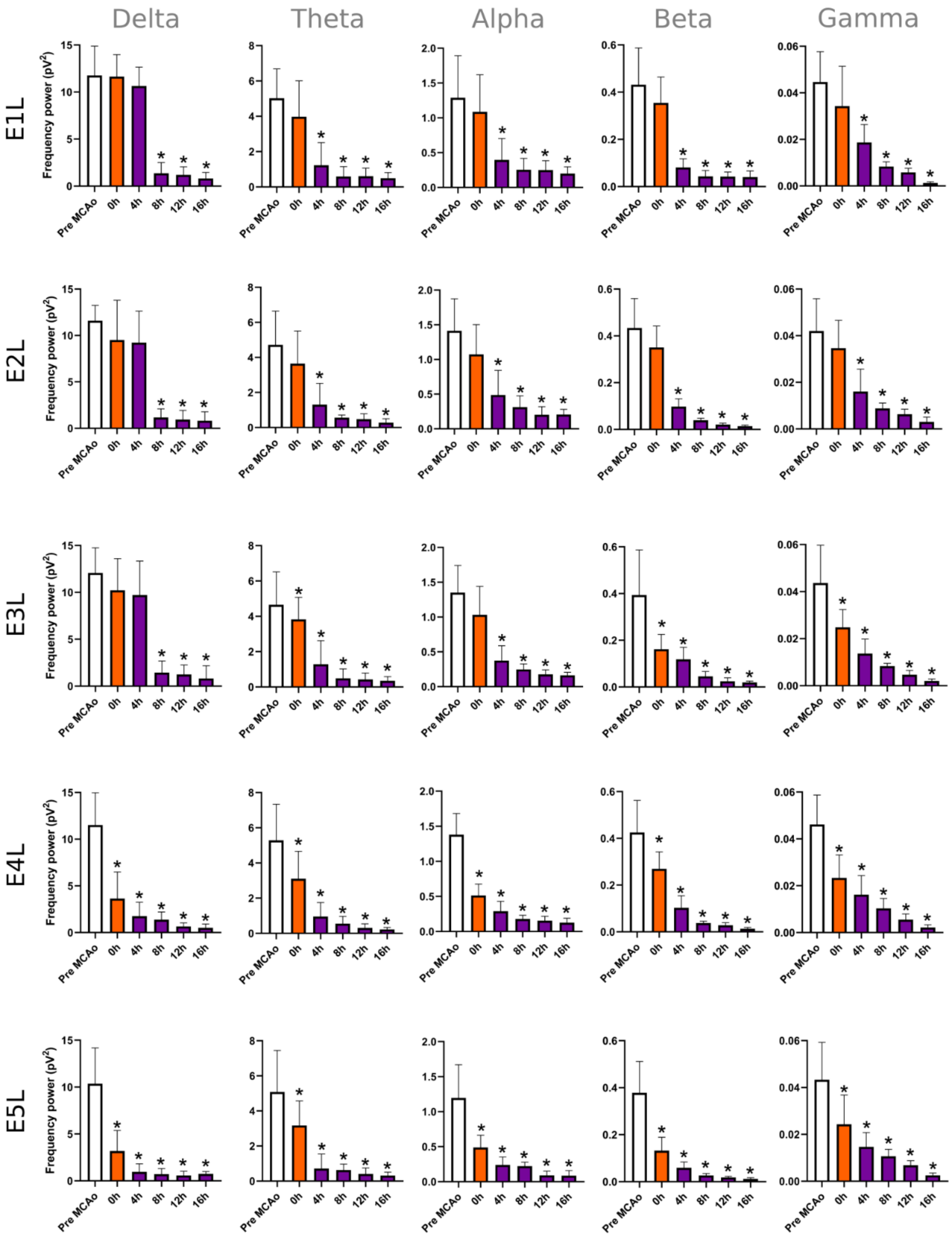

Supplementary Figure 4. Frequency bands in the left electrodes after MCAo in the hypothermia group over time: Insulted hypothermic hemisphere. Mild hypothermia was introduced since the first hour of MCAo and completely established at the fourth hour at 32°C. The 5-min signal segment before MCAo was used as the baseline to compare the changes in frequency bands at 0 h (5 min immediately after), 4 h, 8 h, 12 h, and 16 h after MCAo applying the Wilcoxon matched-pairs signed-rank test. The significant drop in the power spectrum of the frequencies is represented as * (≤ 0.05). MCAo, middle cerebral artery occlusion; pV^2, squared picovolts.

DELTA							
		Pre-MCAo	0h	4h	8h	12h	16h
E1R	M =	10.5586	10.6632	9.8976	10.3916	10.2892	9.8078
	SD =	3.5006	2.5192	4.4400	3.6582	2.9293	3.4143
	T =		8	6	7	9	6
	Z =		-0.135	-0.405	-0.135	-0.405	-0.686
	p =		0.893	0.686	0.893	0.686	0.686
E2R	M =	10.9628	8.9220	8.6064	10.5652	9.1022	9.9288
	SD =	2.8408	3.7555	3.4064	2.2568	3.7774	2.8360
	T =		2	4	6	4	4
	Z =		-1.483	-0.944	-0.405	-0.944	-0.944
	p =		0.138	0.345	0.686	0.345	0.345
E3R	M =	7.9348	8.7956	9.7454	8.9376	7.4610	10.0010
	SD =	2.6349	3.7822	4.2082	2.6296	2.9405	3.4417
	T =		8	14	12	10	12
	Z =		-0.135	-1.753	-1.214	-0.674	-1.214
	p =		0.893	0.08	0.225	0.5	0.225
E4R	M =	8.4142	8.8809	8.5548	8.4774	8.0776	9.2166
	SD =	4.7024	4.4627	3.8701	4.3598	3.6556	4.3020
	T =		9	8	7	7	13
	Z =		-0.405	-0.135	-0.135	-0.135	-1.483
	p =		0.686	0.893	0.893	0.893	0.138
E5R	M =	8.0968	9.0343	10.4388	8.1809	9.3046	8.9750
	SD =	3.8627	3.1846	3.9864	4.2155	4.2503	4.6658
	T =		10	11	8	9	11
	Z =		-0.674	-0.944	-0.135	-0.405	-0.944
	p =		0.5	0.345	0.893	0.686	0.345

THETA							
		Pre-MCAo	0h	4h	8h	12h	16h
E1R	M =	5.1672	4.1452	3.8146	4.3436	5.3484	4.5386
	SD =	2.4523	1.5947	2.5586	2.6053	2.0649	1.9876
	T =		3	4	6	8	3
	Z =		-1.214	-0.944	-0.405	-0.135	-1.214
	p =		0.225	0.345	0.686	0.893	0.225
E2R	M =	4.9680	3.7844	4.2106	3.9600	4.4982	3.9340
	SD =	1.9609	2.2311	3.0136	2.3673	2.5399	2.2098
	T =		4	7	5	7	5
	Z =		-0.944	-0.135	-0.674	-0.135	-0.674
	p =		0.345	0.893	0.5	0.893	0.5
E3R	M =	4.8920	3.0800	3.3684	4.5006	3.4040	3.8998
	SD =	2.7108	2.7246	2.5907	1.9110	1.0810	1.8182
	T =		4	3	8	4	7
	Z =		-0.944	-1.214	-0.135	-0.944	-0.135
	p =		0.345	0.225	0.893	0.345	0.893
E4R	M =	4.6786	4.8710	4.4386	4.7244	4.6758	5.1158
	SD =	1.7047	1.9938	3.2006	1.5643	2.6902	1.4355
	T =		8	6	8	8	8
	Z =		-0.135	-0.405	-0.135	-0.135	-0.135
	p =		0.893	0.686	0.893	0.893	0.893
E5R	M =	4.1782	5.1874	4.3914	4.5038	4.2572	3.4026
	SD =	1.7251	1.9118	1.9756	1.4259	1.7387	1.6217
	T =		9	8	5	8	3
	Z =		-0.405	-0.135	-0.674	-0.135	-1.214
	p =		0.686	0.893	0.5	0.893	0.225

ALPHA							
		Pre-MCAo	0h	4h	8h	12h	16h
E1R	M =	1.2530	1.1462	0.8882	1.1302	1.0744	0.9878
	SD =	0.4035	0.4317	0.5560	0.4188	0.4348	0.4095
	T =		5	3	4	5	4
	Z =		-0.674	-1.214	-0.944	-0.674	-0.944
	p =		0.5	0.225	0.345	0.5	0.345
E2R	M =	1.1128	1.0162	0.9206	0.9656	0.8754	0.9630
	SD =	0.3669	0.3492	0.4567	0.4550	0.5198	0.4893
	T =		5	6	6	5	7
	Z =		-0.674	-0.405	-0.405	-0.674	-0.135
	p =		0.5	0.686	0.686	0.5	0.893
E3R	M =	1.1232	0.8548	0.9076	0.7894	0.8882	0.8310
	SD =	0.2384	0.5203	0.3647	0.4721	0.4762	0.4940
	T =		3	3	4	4	4
	Z =		-1.214	-1.214	-0.944	-0.944	-0.944
	p =		0.225	0.225	0.345	0.345	0.345
E4R	M =	1.1532	1.0370	0.8546	0.8698	1.0524	1.0362
	SD =	0.2638	0.3500	0.4304	0.3225	0.3284	0.3550
	T =		5	3	2	5	8
	Z =		-0.674	-1.214	-1.483	-0.674	-0.135
	p =		0.5	0.225	0.138	0.5	0.893
E5R	M =	1.1926	0.8126	0.8834	0.9624	0.9800	0.9012
	SD =	0.5847	0.5044	0.3349	0.1875	0.3131	0.3203
	T =		5	7	10	10	7
	Z =		-0.674	-0.135	-0.674	-0.674	-0.135
	p =		0.5	0.893	0.5	0.5	0.893

GAMMA							
		Pre-MCAo	0h	4h	8h	12h	16h
E1R	M =	0.0358	0.0370	0.0342	0.0310	0.0354	0.0336
	SD =	0.0178	0.0148	0.0184	0.0184	0.0192	0.0148
	T =		7	4	4	7	6
	Z =		-0.135	-0.365	-0.365	-0.135	-0.405
	p =		0.893	0.715	0.715	0.893	0.686
E2R	M =	0.0364	0.0390	0.0360	0.0346	0.0358	0.0360
	SD =	0.0145	0.0175	0.0187	0.0167	0.0184	0.0177
	T =		8	7	10	6.5	8.5
	Z =		-0.135	-0.135	-0.674	-0.271	-0.271
	p =		0.892	0.893	0.5	0.786	0.786
E3R	M =	0.0374	0.0360	0.0376	0.0352	0.0330	0.0390
	SD =	0.0169	0.0168	0.0184	0.0158	0.0169	0.0160
	T =		8	7	6	3	8.5
	Z =		-0.135	-0.135	-0.405	-1.219	-0.271
	p =		0.892	0.893	0.686	0.223	0.786
E4R	M =	0.0368	0.0342	0.0370	0.0296	0.0414	0.0370
	SD =	0.0158	0.0182	0.0176	0.0166	0.0146	0.0180
	T =		7	8.5	3	8.5	9
	Z =		-0.135	-0.271	-0.736	-0.272	-0.405
	p =		0.893	0.786	0.461	0.785	0.686
E5R	M =	0.0346	0.0372	0.0380	0.0340	0.0420	0.0390
	SD =	0.0170	0.0181	0.0114	0.0185	0.0163	0.0183
	T =		9	10	8	10	9
	Z =		-0.405	-0.674	-0.135	-0.674	-0.405
	p =		0.686	0.5	0.893	0.5	0.686

BETA							
		Pre-MCAo	0h	4h	8h	12h	16h
E1R	M =	0.3328	0.2942	0.3326	0.3300	0.3152	0.3028
	SD =	0.1614	0.2157	0.1790	0.1663	0.1829	0.1722
	T =		7	5	8	10	7
	Z =		-0.135	-0.674	-0.405	-0.674	-0.135
	p =		0.893	0.5	0.686	0.5	0.893
E2R	M =	0.4198	0.3766	0.4290	0.3548	0.3718	0.4500
	SD =	0.1768	0.1797	0.1235	0.1560	0.1624	0.1409
	T =		6	8	7	6	9
	Z =		-0.405	-0.135	-0.135	-0.405	-0.405
	p =		0.686	0.893	0.893	0.686	0.686
E3R	M =	0.3794	0.3648	0.3204	0.3914	0.4114	0.3904
	SD =	0.1723	0.2005	0.1580	0.1720	0.1460	0.1966
	T =		7	6	7	9	9
	Z =		-0.135	-0.405	-0.135	-0.405	-0.405
	p =		0.893	0.686	0.893	0.686	0.686
E4R	M =	0.3628	0.3434	0.3932	0.3338	0.3346	0.3510
	SD =	0.2073	0.1333	0.1724	0.1351	0.1282	0.1560
	T =		7	9	6	7	6
	Z =		-0.135	-0.405	-0.405	-0.135	-0.405
	p =		0.893	0.686	0.686	0.892	0.686
E5R	M =	0.3254	0.3534	0.2752	0.3768	0.3570	0.3420
	SD =	0.1425	0.1752	0.2312	0.1544	0.1505	0.1629
	T =		9	5	10	12	8
	Z =		-0.405	-0.674	-0.674	-1.214	-0.135
	p =		0.686	0.5	0.5	0.225	0.893

Supplementary Table 7. Statistical analysis report - Changes in the frequency bands in the right electrodes after MCAo in the control group: Healthy normothermic hemisphere. The Wilcoxon matched-pairs signed-rank test was applied to observe how the power spectra of the frequency bands changed 5 min after MCAo (0 h), at 4, 8, and 12 h, using the frequency bands collected 5 min prior to the MCAo as a baseline from the left injured hemisphere of normothermic brains. The statistically significant drop in the power spectra of the frequency bands was at ≤ 0.05. M, mean; SD, Standard deviation; T, t-test statistic; z, z score.

DELTA

	Pre-MCAo	0h	4h	8h	12h	16h
E1L	M = 10.4682	M = 10.0068	M = 10.1638	M = 9.8360	M = 8.8888	M = 1.6788
	SD = 5.5663	SD = 5.0480	SD = 4.0827	SD = 3.4844	SD = 3.3584	SD = 1.0266
		T = 3	T = 6	T = 5	T = 3	T = 0
		Z = -1.214	Z = -0.405	Z = -0.674	Z = -1.214	Z = -2.023
		p = 0.225	p = 0.686	p = 0.5	p = 0.225	p = **0.043**
E2L	M = 9.2506	M = 8.0172	M = 7.8132	M = 7.7328	M = 7.4852	M = 1.2540
	SD = 3.1210	SD = 3.6062	SD = 1.7118	SD = 1.8217	SD = 2.0187	SD = 0.8154
		T = 1	T = 3	T = 4	T = 1	T = 0
		Z = -1.753	Z = -1.214	Z = -0.944	Z = -1.753	Z = -2.023
		p = 0.08	p = 0.225	p = 0.345	p = 0.08	p = **0.043**
E3L	M = 10.0810	M = 9.1466	M = 1.6598	M = 1.1514	M = 0.7356	M = 0.5426
	SD = 4.7992	SD = 3.1421	SD = 0.4796	SD = 0.2865	SD = 0.1397	SD = 0.1170
		T = 4	T = 0	T = 0	T = 0	T = 0
		Z = -0.944	Z = -2.023	Z = -2.023	Z = -2.023	Z = -2.023
		p = 0.345	p = **0.043**	p = **0.043**	p = **0.043**	p = **0.043**
E4L	M = 12.3094	M = 3.7782	M = 2.0844	M = 0.7024	M = 0.6132	M = 0.4476
	SD = 2.5814	SD = 3.3659	SD = 1.8004	SD = 0.5184	SD = 0.3475	SD = 0.2053
		T = 0	T = 0	T = 0	T = 0	T = 0
		Z = -2.023	Z = -2.023	Z = -2.023	Z = -2.023	Z = -2.023
		p = **0.043**	p = **0.043**	p = **0.043**	p = **0.043**	p = **0.043**
E5L	M = 15.2946	M = 7.0786	M = 2.0646	M = 1.2094	M = 1.0596	M = 1.1092
	SD = 3.5788	SD = 3.3708	SD = 2.2009	SD = 1.9767	SD = 1.7971	SD = 1.1773
		T = 0	T = 0	T = 0	T = 0	T = 0
		Z = -2.023	Z = -2.023	Z = -2.023	Z = -2.023	Z = -2.023
		p = **0.043**	p = **0.043**	p = **0.043**	p = **0.043**	p = **0.043**

THETA

	Pre-MCAo	0h	4h	8h	12h	16h
E1L	M = 6.1898	M = 5.6374	M = 5.6858	M = 5.1074	M = 1.7166	M = 0.9498
	SD = 2.3230	SD = 2.6932	SD = 1.9629	SD = 1.8544	SD = 1.2233	SD = 0.7129
		T = 7	T = 5	T = 2	T = 0	T = 0
		Z = -0.135	Z = -.674	Z = -1.483	Z = -2.023	Z = -2.023
		p = 0.893	p = 0.5	p = 0.138	p = **0.043**	p = **0.043**
E2L	M = 4.3120	M = 4.1374	M = 3.7702	M = 3.4996	M = 0.6052	M = 0.043
	SD = 2.6088	SD = 1.8495	SD = 1.6179	SD = 1.2234	SD = 0.2755	SD = 0.043
		T = 5	T = 4	T = 3	T = 0	T = 0.043
		Z = -0.674	Z = -.944	Z = -1.214	Z = -2.023	Z = 0.043
		p = 0.5	p = 0.345	p = 0.225	p = **0.043**	p = **0.043**
E3L	M = 4.5930	M = 4.0644	M = 0.4668	M = 0.4546	M = 0.4920	M = 0.043
	SD = 1.1382	SD = 1.1613	SD = 0.3300	SD = 0.3149	SD = 0.3062	SD = 0.043
		T = 2	T = 0	T = 0	T = 0	T = 0.043
		Z = -1.483	Z = -2.023	Z = -2.023	Z = -2.023	Z = 0.043
		p = 0.138	p = **0.043**	p = **0.043**	p = **0.043**	p = **0.043**
E4L	M = 5.8200	M = 1.9146	M = 0.5980	M = 0.4930	M = 0.5038	M = 0.043
	SD = 2.1530	SD = 1.2094	SD = 0.2395	SD = 0.2667	SD = 0.2392	SD = 0.043
		T = 0	T = 0	T = 0	T = 0	T = 0.043
		Z = -2.023	Z = -2.023	Z = -2.023	Z = -2.023	Z = 0.043
		p = **0.043**	p = **0.043**	p = **0.043**	p = **0.043**	p = **0.043**
E5L	M = 7.0586	M = 3.3922	M = 0.4640	M = 0.4574	M = 0.3494	M = 0.043
	SD = 2.1950	SD = 1.3011	SD = 0.2900	SD = 0.2246	SD = 0.2084	SD = 0.043
		T = 0	T = 0	T = 0	T = 0	T = 0.043
		Z = -2.023	Z = -2.023	Z = -2.023	Z = -2.023	Z = 0.043
		p = **0.043**	p = **0.043**	p = **0.043**	p = **0.043**	p = **0.043**

ALPHA

	Pre-MCAo	0h	4h	8h	12h	16h
E1L	M = 1.3096	M = 1.1284	M = 1.0842	M = 1.0352	M = 0.1778	M = 0.1234
	SD = 0.4639	SD = 0.4390	SD = 0.4922	SD = 0.4135	SD = 0.1289	SD = 0.0708
		T = 3	T = 3	T = 1	T = 0	T = 0
		Z = -1.214	Z = -1.214	Z = -1.753	Z = -2.023	Z = -2.023
		p = 0.225	p = 0.225	p = 0.08	p = **0.043**	p = **0.043**
E2L	M = 1.2880	M = 1.0894	M = 0.1760	M = 0.1246	M = 0.1222	M = 0.0786
	SD = 0.3752	SD = 0.5218	SD = 0.0602	SD = 0.0208	SD = 0.0354	SD = 0.0413
		T = 2	T = 0	T = 0	T = 0	T = 0
		Z = -1.483	Z = -2.023	Z = -2.023	Z = -2.023	Z = -2.023
		p = 0.138	p = **0.043**	p = **0.043**	p = **0.043**	p = **0.043**
E3L	M = 1.7850	M = 1.5468	M = 0.2578	M = 0.2360	M = 0.2042	M = 0.1598
	SD = 0.8414	SD = 0.8695	SD = 0.0872	SD = 0.0581	SD = 0.0727	SD = 0.0518
		T = 3	T = 0	T = 0	T = 0	T = 0
		Z = -1.214	Z = -2.023	Z = -2.023	Z = -2.023	Z = -2.023
		p = 0.225	p = **0.043**	p = **0.043**	p = **0.043**	p = **0.043**
E4L	M = 2.2130	M = 0.6960	M = 0.3040	M = 0.2900	M = 0.2346	M = 0.2240
	SD = 0.9940	SD = 0.3363	SD = 0.2523	SD = 0.1219	SD = 0.1730	SD = 0.0770
		T = 0	T = 0	T = 0	T = 0	T = 0
		Z = -2.023	Z = -2.023	Z = -2.023	Z = -2.023	Z = -2.023
		p = **0.043**	p = **0.043**	p = **0.043**	p = **0.043**	p = **0.043**
E5L	M = 2.5980	M = 0.7778	M = 0.2420	M = 0.1966	M = 0.1820	M = 0.1334
	SD = 1.0942	SD = 0.3482	SD = 0.0455	SD = 0.0651	SD = 0.0581	SD = 0.0619
		T = 0	T = 0	T = 0	T = 0	T = 0
		Z = -2.023	Z = -2.023	Z = -2.023	Z = -2.023	Z = -2.023
		p = **0.043**	p = **0.043**	p = **0.043**	p = **0.043**	p = **0.043**

BETA

	Pre-MCAo	0h	4h	8h	12h	16h
E1L	M = 0.3808	M = 0.3662	M = 0.3300	M = 0.2990	M = 0.0698	M = 0.0442
	SD = 0.1689	SD = 0.1215	SD = 0.1201	SD = 0.1088	SD = 0.0181	SD = 0.0211
		T = 7	T = 5	T = 5	T = 0	T = 0
		Z = -0.135	Z = -0.674	Z = -.674	Z = -2.023	Z = -2.023
		p = 0.893	p = 0.5	p = 0.5	p = **0.043**	p = **0.043**
E2L	M = 0.3218	M = 0.1104	M = 0.3476	M = 0.1154	M = 0.0992	M = 0.2942
	SD = 0.2190	SD = 0.0208	SD = 0.1581	SD = 0.0259	SD = 0.0234	SD = 0.1901
		T = 0	T = 11	T = 0	T = 0	T = 6
		Z = -2.023	Z = -0.944	Z = -2.023	Z = -2.023	Z = -0.405
		p = **0.043**	p = 0.345	p = **0.043**	p = **0.043**	p = 0.686
E3L	M = 0.3638	M = 0.1088	M = 0.0538	M = 0.0444	M = 0.0190	M = 0.0150
	SD = 0.2125	SD = 0.0370	SD = 0.0364	SD = 0.0268	SD = 0.0048	SD = 0.0055
		T = 0	T = 0	T = 0	T = 0	T = 0
		Z = -2.023	Z = -2.023	Z = -2.023	Z = -2.023	Z = -2.023
		p = **0.043**	p = **0.043**	p = **0.043**	p = **0.043**	p = **0.043**
E4L	M = 0.5822	M = 0.3106	M = 0.5654	M = 0.2946	M = 0.2988	M = 0.4830
	SD = 0.2619	SD = 0.0228	SD = 0.1854	SD = 0.0161	SD = 0.0170	SD = 0.1237
		T = 0	T = 7	T = 0	T = 0	T = 5
		Z = -2.023	Z = -0.135	Z = -2.023	Z = -2.023	Z = -0.674
		p = **0.043**	p = 0.893	p = **0.043**	p = **0.043**	p = 0.5
E5L	M = 0.4152	M = 0.1330	M = 0.0410	M = 0.0350	M = 0.0300	M = 0.0340
	SD = 0.2242	SD = 0.0853	SD = 0.0156	SD = 0.0219	SD = 0.0216	SD = 0.0147
		T = 0	T = 0	T = 0	T = 0	T = 0
		Z = -2.023	Z = -2.023	Z = -2.023	Z = -2.023	Z = -2.023
		p = **0.043**	p = **0.043**	p = **0.043**	p = **0.043**	p = **0.043**

GAMMA

	Pre-MCAo	0h	4h	8h	12h	16h
E1L	M = 0.0392	M = 0.0404	M = 0.0382	M = 0.0364	M = 0.0328	M = 0.0142
	SD = 0.0265	SD = 0.0186	SD = 0.0181	SD = 0.0177	SD = 0.0169	SD = 0.0049
		T = 9	T = 7	T = 5	T = 3	T = 0
		Z = -0.405	Z = -0.136	Z = -0.674	Z = -1.214	Z = -2.032
		p = 0.686	p = 0.892	p = 0.5	p = 0.225	p = **0.042**
E2L	M = 0.0438	M = 0.0446	M = 0.0428	M = 0.0404	M = 0.0392	M = 0.0152
	SD = 0.0235	SD = 0.0146	SD = 0.0168	SD = 0.0167	SD = 0.0167	SD = 0.0069
		T = 8.5	T = 6	T = 4	T = 3.5	T = 0
		Z = -0.272	Z = -0.406	Z = -0.944	Z = -1.084	Z = -2.023
		p = 0.785	p = 0.684	p = 0.345	p = 0.279	p = **0.043**
E3L	M = 0.0466	M = 0.0166	M = 0.0078	M = 0.0064	M = 0.0054	M = 0.0024
	SD = 0.0206	SD = 0.0090	SD = 0.0016	SD = 0.0015	SD = 0.0018	SD = 0.0013
		T = 0	T = 0	T = 0	T = 0	T = 0
		Z = -2.023	Z = -2.023	Z = -2.023	Z = -2.023	Z = -2.023
		p = **0.043**	p = **0.043**	p = **0.043**	p = **0.043**	p = **0.043**
E4L	M = 0.0494	M = 0.0232	M = 0.0094	M = 0.0060	M = 0.0044	M = 0.0023
	SD = 0.0257	SD = 0.0140	SD = 0.0038	SD = 0.0023	SD = 0.0028	SD = 0.0010
		T = 0	T = 0	T = 0	T = 0	T = 0
		Z = -2.023	Z = -2.023	Z = -2.023	Z = -2.023	Z = -2.023
		p = **0.043**	p = **0.043**	p = **0.043**	p = **0.043**	p = **0.043**
E5L	M = 0.0548	M = 0.0258	M = 0.0102	M = 0.0066	M = 0.0054	M = 0.0032
	SD = 0.0168	SD = 0.0107	SD = 0.0028	SD = 0.0021	SD = 0.0015	SD = 0.0015
		T = 0	T = 0	T = 0	T = 0	T = 0
		Z = -2.023	Z = -2.023	Z = -2.023	Z = -2.023	Z = -2.023
		p = **0.043**	p = **0.043**	p = **0.043**	p = **0.043**	p = **0.043**

Supplementary Table 8. Statistical analysis report - Changes in the frequency bands in the left electrodes after MCAo in the control group: Insulted normothermic hemisphere. The Wilcoxon matched-pairs signed-rank test was applied to observe how the power spectra of the frequency bands changed 5 min after MCAo (0 h), at 4, 8, and 12 h, using the frequency bands collected 5 min prior to the MCAo as a baseline from the left injured hemisphere of normothermic brains. The statistically significant drop in the power spectra of the frequency bands was at ≤ 0.05. M, mean; SD, Standard deviation; T, t-test statistic; z, z score.

DELTA

	Pre-MCAo	0h	4h	8h	12h	16h
E1R	M = 11.3482	M = 10.4710	M = 10.8623	M = 9.5560	M = 5.7725	M = 2.7745
	SD = 1.6941	SD = 1.8773	SD = 1.8926	SD = 1.1093	SD = 1.1662	SD = 0.7001
		T = 7	T = 10	T = 1	T = 0	T = 0
		Z = -0.734	Z = -0.105	Z = -1.992	Z = -2.201	Z = -2.201
		p = 0.463	p = 0.917	p = **0.046**	p = **0.028**	p = **0.028**
E2R	M = 11.5612	M = 10.8065	M = 11.0110	M = 9.8495	M = 5.1592	M = 2.8643
	SD = 2.1134	SD = 2.0780	SD = 1.8981	SD = 1.2922	SD = 0.8626	SD = 0.6718
		T = 9	T = 6	T = 4	T = 0	T = 0
		Z = -0.314	Z = -0.943	Z = -1.363	Z = -2.201	Z = -2.201
		p = 0.753	p = 0.345	p = 0.173	p = **0.028**	p = **0.028**
E3R	M = 11.7882	M = 11.4600	M = 11.0258	M = 9.6347	M = 5.3612	M = 2.3927
	SD = 1.9443	SD = 1.4588	SD = 1.6873	SD = 1.1530	SD = 0.6652	SD = 0.5384
		T = 8	T = 6	T = 3	T = 0	T = 0
		Z = -0.524	Z = -0.943	Z = -1.572	Z = -2.201	Z = -2.201
		p = 0.6	p = 0.345	p = 0.116	p = **0.028**	p = **0.028**
E4R	M = 11.4932	M = 10.9417	M = 10.7275	M = 8.9727	M = 5.2567	M = 2.1950
	SD = 1.6979	SD = 1.3767	SD = 1.6443	SD = 1.0301	SD = 0.9729	SD = 0.7380
		T = 7	T = 7	T = 1	T = 0	T = 0
		Z = -0.734	Z = -0.734	Z = -1.992	Z = -2.201	Z = -2.201
		p = 0.463	p = 0.463	p = **0.046**	p = **0.028**	p = **0.028**
E5R	M = 11.3087	M = 11.0185	M = 10.7923	M = 8.8525	M = 5.5450	M = 1.8100
	SD = 2.2322	SD = 1.7061	SD = 1.4939	SD = 1.1638	SD = 1.0020	SD = 0.9538
		T = 10	T = 6	T = 2	T = 0	T = 0
		Z = -0.105	Z = -0.943	Z = -1.782	Z = -2.201	Z = -2.201
		p = 0.917	p = 0.345	p = 0.075	p = **0.028**	p = **0.028**

THETA

	Pre-MCAo	0h	4h	8h	12h	16h
E1R	M = 6.0997	M = 5.7672	M = 3.9103	M = 1.0153	M = 0.5753	M = 0.1842
	SD = 1.8408	SD = 1.9593	SD = 1.1614	SD = 0.2508	SD = 0.1063	SD = 0.0630
		T = 10	T = 1	T = 0	T = 0	T = 0
		Z = -0.105	Z = -1.992	Z = -2.201	Z = -2.201	Z = -2.201
		p = 0.917	p = **0.046**	p = **0.028**	p = **0.028**	p = **0.028**
E2R	M = 5.7457	M = 5.8085	M = 3.3545	M = 0.8948	M = 0.5508	M = 0.1867
	SD = 1.2341	SD = 1.0330	SD = 0.7756	SD = 0.2065	SD = 0.0891	SD = 0.0354
		T = 14	T = 0	T = 0	T = 0	T = 0
		Z = -0.734	Z = -2.201	Z = -2.201	Z = -2.201	Z = -2.201
		p = 0.463	p = **0.028**	p = **0.028**	p = **0.028**	p = **0.028**
E3R	M = 6.1647	M = 5.8688	M = 4.5803	M = 1.0365	M = 0.4700	M = 0.1793
	SD = 1.0469	SD = 0.9917	SD = 0.4810	SD = 0.2490	SD = 0.0944	SD = 0.0462
		T = 6	T = 0	T = 0	T = 0	T = 0
		Z = -0.943	Z = -2.201	Z = -2.201	Z = -2.201	Z = -2.201
		p = 0.345	p = **0.028**	p = **0.028**	p = **0.028**	p = **0.028**
E4R	M = 6.1687	M = 5.5432	M = 4.4592	M = 1.0110	M = 0.5968	M = 0.1840
	SD = 0.8205	SD = 5.8065	SD = 0.7252	SD = 0.2925	SD = 0.1461	SD = 0.0409
		T = 6	T = 1	T = 0	T = 0	T = 0
		Z = -0.943	Z = -1.992	Z = -2.201	Z = -2.201	Z = -2.201
		p = 0.345	p = **0.028**	p = **0.028**	p = **0.028**	p = **0.028**
E5R	M = 5.6583	M = 5.8065	M = 4.5615	M = 0.9745	M = 0.5187	M = 0.1612
	SD = 0.9319	SD = 0.6864	SD = 0.5928	SD = 0.1719	SD = 0.1117	SD = 0.0549
		T = 12	T = 1	T = 0	T = 0	T = 0
		Z = -0.314	Z = -1.992	Z = -2.201	Z = -2.201	Z = -2.201
		p = 0.753	p = **0.046**	p = **0.028**	p = **0.028**	p = **0.028**

ALPHA

	Pre-MCAo	0h	4h	8h	12h	16h
E1R	M = 0.9808	M = 0.8618	M = 0.2683	M = 0.0706	M = 0.0365	M = 0.0151
	SD = 0.1852	SD = 0.1629	SD = 0.0971	SD = 0.0263	SD = 0.0204	SD = 0.0066
		T = 5	T = 0	T = 0	T = 0	T = 0
		Z = -1.153	Z = -2.201	Z = -2.201	Z = -2.201	Z = -2.201
		p = 0.249	p = **0.028**	p = **0.028**	p = **0.028**	p = **0.028**
E2R	M = 0.9973	M = 0.9152	M = 0.4212	M = 0.0788	M = 0.0332	M = 0.0193
	SD = 0.1639	SD = 0.1737	SD = 0.0798	SD = 0.0116	SD = 0.0092	SD = 0.0103
		T = 2	T = 0	T = 0	T = 0	T = 0
		Z = -1.782	Z = -2.201	Z = -2.201	Z = -2.201	Z = -2.201
		p = 0.075	p = **0.028**	p = **0.028**	p = **0.028**	p = **0.028**
E3R	M = 0.9470	M = 0.9690	M = 0.4402	M = 0.0900	M = 0.0102	M = 0.0215
	SD = 0.1115	SD = 0.0931	SD = 0.0728	SD = 0.0096	SD = 0.0102	SD = 0.0129
		T = 11.5	T = 0	T = 0	T = 0	T = 0
		Z = -0.21	Z = -2.201	Z = -2.201	Z = -2.201	Z = -2.201
		p = 0.833	p = **0.028**	p = **0.028**	p = **0.028**	p = **0.028**
E4R	M = 1.3262	M = 0.9752	M = 0.4467	M = 0.1957	M = 0.0893	M = 0.0509
	SD = 0.8543	SD = 0.0948	SD = 0.0476	SD = 0.0612	SD = 0.0401	SD = 0.0386
		T = 8	T = 0	T = 0	T = 0	T = 0
		Z = -0.524	Z = -2.201	Z = -2.201	Z = -2.201	Z = -2.201
		p = 0.6	p = **0.028**	p = **0.028**	p = **0.028**	p = **0.028**
E5R	M = 1.2093	M = 0.9747	M = 0.3533	M = 0.2058	M = 0.0782	M = 0.0485
	SD = 0.4165	SD = 0.0759	SD = 0.0862	SD = 0.0497	SD = 0.0147	SD = 0.0101
		T = 12	T = 0	T = 0	T = 0	T = 0
		Z = -0.315	Z = -2.201	Z = -2.201	Z = -2.201	Z = -2.201
		p = 0.752	p = **0.028**	p = **0.028**	p = **0.028**	p = **0.028**

BETA

	Pre-MCAo	0h	4h	8h	12h	16h
E1R	M = 0.3627	M = 0.3777	M = 0.2467	M = 0.0850	M = 0.0440	M = 0.0153
	SD = 0.1061	SD = 0.0990	SD = 0.0493	SD = 0.0296	SD = 0.0126	SD = 0.0066
		T = 12	T = 1	T = 0	T = 0	T = 0
		Z = -0.314	Z = -1.992	Z = -2.201	Z = -2.201	Z = -2.201
		p = 0.753	p = **0.046**	p = **0.028**	p = **0.028**	p = **0.027**
E2R	M = 0.4400	M = 0.4713	M = 0.2923	M = 0.1133	M = 0.0778	M = 0.0357
	SD = 0.1099	SD = 0.0854	SD = 0.0453	SD = 0.0203	SD = 0.0149	SD = 0.0066
		T = 13	T = 1	T = 0	T = 0	T = 0
		Z = -0.524	Z = -1.992	Z = -2.201	Z = -2.201	Z = -2.201
		p = 0.6	p = **0.046**	p = **0.028**	p = **0.028**	p = **0.028**
E3R	M = 0.4128	M = 0.4237	M = 0.2692	M = 0.1268	M = 0.0562	M = 0.0157
	SD = 0.0887	SD = 0.0703	SD = 0.0324	SD = 0.0273	SD = 0.0140	SD = 0.0092
		T = 12	T = 0	T = 0	T = 0	T = 0
		Z = -0.314	Z = -2.201	Z = -2.201	Z = -2.201	Z = -2.201
		p = 0.753	p = **0.028**	p = **0.028**	p = **0.028**	p = **0.028**
E4R	M = 0.4653	M = 0.4917	M = 0.2668	M = 0.0958	M = 0.0380	M = 0.0103
	SD = 0.0965	SD = 0.0816	SD = 0.0637	SD = 0.0193	SD = 0.0060	SD = 0.0037
		T = 15	T = 0	T = 0	T = 0	T = 0
		Z = -0.943	Z = -2.201	Z = -2.201	Z = -2.201	Z = -2.201
		p = 0.345	p = **0.028**	p = **0.028**	p = **0.028**	p = **0.028**
E5R	M = 0.4458	M = 0.4613	M = 0.2168	M = 0.0843	M = 0.0350	M = 0.0127
	SD = 0.0676	SD = 0.0474	SD = 0.0300	SD = 0.0197	SD = 0.0084	SD = 0.0073
		T = 14	T = 0	T = 0	T = 0	T = 0
		Z = -0.734	Z = -2.201	Z = -2.201	Z = -2.201	Z = -2.201
		p = 0.463	p = **0.028**	p = **0.028**	p = **0.028**	p = **0.028**

GAMMA

	Pre-MCAo	0h	4h	8h	12h	16h
E1R	M = 0.0460	M = 0.0467	M = 0.0157	M = 0.0082	M = 0.0042	M = 0.0013
	SD = 0.0058	SD = 0.0070	SD = 0.0031	SD = 0.0028	SD = 0.0018	SD = 0.0009
		T = 13	T = 0	T = 0	T = 0	T = 0
		Z = -0.526	Z = -2.201	Z = -2.201	Z = -2.201	Z = -2.201
		p = 0.599	p = **0.028**	p = **0.028**	p = **0.028**	p = **0.028**
E2R	M = 0.0455	M = 0.0437	M = 0.0168	M = 0.0098	M = 0.0041	M = 0.0012
	SD = 0.0058	SD = 0.0090	SD = 0.0046	SD = 0.0018	SD = 0.0014	SD = 0.0007
		T = 6	T = 0	T = 0	T = 0	T = 0
		Z = -0.946	Z = -2.207	Z = -2.201	Z = -2.201	Z = -2.201
		p = 0.344	p = **0.027**	p = **0.028**	p = **0.028**	p = **0.028**
E3R	M = 0.0468	M = 0.0493	M = 0.0168	M = 0.0096	M = 0.0041	M = 0.0010
	SD = 0.0089	SD = 0.0073	SD = 0.0035	SD = 0.0020	SD = 0.0010	SD = 0.0005
		T = 13	T = 0	T = 0	T = 0	T = 0
		Z = -0.524	Z = -2.207	Z = -2.201	Z = -2.201	Z = -2.201
		p = 0.6	p = **0.028**	p = **0.028**	p = **0.028**	p = **0.028**
E4R	M = 0.0512	M = 0.0513	M = 0.0180	M = 0.0107	M = 0.0051	M = 0.0013
	SD = 0.0079	SD = 0.0069	SD = 0.0050	SD = 0.0039	SD = 0.0020	SD = 0.0005
		T = 11	T = 0	T = 0	T = 0	T = 0
		Z = -0.105	Z = -2.207	Z = -2.207	Z = -2.201	Z = -2.201
		p = 0.916	p = **0.027**	p = **0.027**	p = **0.028**	p = **0.028**
E5R	M = 0.0522	M = 0.0505	M = 0.0172	M = 0.0098	M = 0.0040	M = 0.0008
	SD = 0.0061	SD = 0.0059	SD = 0.0044	SD = 0.0036	SD = 0.0011	SD = 0.0005
		T = 6	T = 0	T = 0	T = 0	T = 0
		Z = -0.405	Z = -2.201	Z = -2.201	Z = -2.201	Z = -2.201
		p = 0.686	p = **0.028**	p = **0.028**	p = **0.028**	p = **0.028**

Supplementary Table 9. Statistical analysis report - Changes in the frequency bands in the right electrodes after MCAo in the hypothermia group: Healthy hypothermic hemisphere. The Wilcoxon matched-pairs signed-rank test was applied to observe how the power spectra of the frequency bands changed 5 min after MCAo (0 h), at 4, 8, and 12 h, using the frequency bands collected 5 min prior to the MCAo as a baseline from the left injured hemisphere of normothermic brains. The statistically significant drop in the power spectra of the frequency bands was at ≤ 0.05. M, mean; SD, Standard deviation; T, t-test statistic; z, z score.

DELTA						
	Pre-MCAo	0h	4h	8h	12h	16h
E1L	M = 11.7717	M = 11.6402	M = 10.6383	M = 1.3690	M = 1.1992	M = 0.8173
	SD = 3.1128	SD = 2.3445	SD = 2.0129	SD = 1.1380	SD = 0.8543	SD = 0.6396
		T = 10	T = 6	T = 0	T = 0	T = 0
		Z = -0.105	Z = -0.943	Z = -2.201	Z = -2.201	Z = -2.201
		p = 0.917	p = 0.345	p = **0.028**	p = **0.028**	p = **0.028**
E2L	M = 11.5968	M = 9.5233	M = 9.2333	M = 1.1662	M = 0.9467	M = 0.8203
	SD = 1.6497	SD = 4.2670	SD = 3.4007	SD = 0.9256	SD = 0.9906	SD = 0.9623
		T = 6	T = 2	T = 0	T = 0	T = 0
		Z = -0.943	Z = -1.782	Z = -2.201	Z = -2.201	Z = -2.201
		p = 0.345	p = 0.075	p = **0.028**	p = **0.028**	p = **0.028**
E3L	M = 12.0628	M = 10.2143	M = 9.7148	M = 1.4415	M = 1.2403	M = 0.8180
	SD = 2.6900	SD = 3.3978	SD = 3.6344	SD = 1.2442	SD = 1.0194	SD = 1.3485
		T = 3	T = 3	T = 0	T = 0	T = 0
		Z = -1.572	Z = -1.572	Z = -2.201	Z = -2.201	Z = -2.201
		p = 0.116	p = 0.116	p = **0.028**	p = **0.028**	p = **0.028**
E4L	M = 11.5200	M = 3.6485	M = 1.7558	M = 1.3757	M = 0.6517	M = 0.5077
	SD = 3.4648	SD = 2.8508	SD = 1.4850	SD = 0.8257	SD = 0.3775	SD = 0.4140
		T = 0	T = 0	T = 0	T = 0	T = 0
		Z = -2.201	Z = -2.201	Z = -2.201	Z = -2.201	Z = -2.201
		p = **0.028**	p = **0.028**	p = **0.028**	p = **0.028**	p = **0.028**
E5L	M = 10.3720	M = 3.1838	M = 0.9603	M = 0.7150	M = 0.5788	M = 0.7425
	SD = 3.8115	SD = 2.1848	SD = 0.8591	SD = 0.5914	SD = 0.4586	SD = 0.2568
		T = 0	T = 0	T = 0	T = 0	T = 0
		Z = -2.201	Z = -2.201	Z = -2.201	Z = -2.201	Z = -2.201
		p = **0.028**	p = **0.028**	p = **0.028**	p = **0.028**	p = **0.028**

THETA						
	Pre-MCAo	0h	4h	8h	12h	16h
E1L	M = 5.0202	M = 3.9763	M = 1.2312	M = 0.5877	M = 0.6072	M = 0.4945
	SD = 1.6638	SD = 2.0358	SD = 1.2781	SD = 0.5609	SD = 0.4581	SD = 0.3192
		T = 4	T = 0	T = 0	T = 0	T = 0
		Z = -1.363	Z = -2.201	Z = -2.201	Z = -2.201	Z = -2.201
		p = 0.173	p = **0.028**	p = **0.028**	p = **0.028**	p = **0.028**
E2L	M = 4.7170	M = 3.6497	M = 1.3097	M = 0.5620	M = 0.4798	M = 0.2785
	SD = 1.9234	SD = 1.8614	SD = 1.2123	SD = 0.1580	SD = 0.3042	SD = 0.2278
		T = 8	T = 0	T = 0	T = 0	T = 0
		Z = -0.524	Z = -2.201	Z = -2.201	Z = -2.201	Z = -2.201
		p = 0.6	p = **0.028**	p = **0.028**	p = **0.028**	p = **0.028**
E3L	M = 4.6558	M = 3.8293	M = 1.2933	M = 0.4905	M = 0.4293	M = 0.3507
	SD = 1.8585	SD = 1.2479	SD = 1.3283	SD = 0.5333	SD = 0.3633	SD = 0.2418
		T = 1	T = 1	T = 0	T = 0	T = 0
		Z = -1.992	Z = -1.992	Z = -2.201	Z = -2.201	Z = -2.201
		p = **0.046**	p = **0.046**	p = **0.028**	p = **0.028**	p = **0.028**
E4L	M = 5.2928	M = 2.7698	M = 0.9542	M = 0.5473	M = 0.3133	M = 0.2223
	SD = 2.0406	SD = 1.1853	SD = 0.7966	SD = 0.4166	SD = 0.2323	SD = 0.1120
		T = 1	T = 0	T = 0	T = 0	T = 0
		Z = -1.992	Z = -2.201	Z = -2.201	Z = -2.201	Z = -2.201
		p = **0.046**	p = **0.028**	p = **0.028**	p = **0.028**	p = **0.028**
E5L	M = 5.0803	M = 3.1733	M = 0.7157	M = 0.6293	M = 0.4095	M = 0.3150
	SD = 2.3683	SD = 1.3936	SD = 0.8308	SD = 0.3394	SD = 0.3403	SD = 0.1939
		T = 0	T = 0	T = 0	T = 0	T = 0
		Z = -2.201	Z = -2.201	Z = -2.201	Z = -2.201	Z = -2.201
		p = **0.028**	p = **0.028**	p = **0.028**	p = **0.028**	p = **0.028**

ALPHA						
	Pre-MCAo	0h	4h	8h	12h	16h
E1L	M = 1.2895	M = 1.0873	M = 0.3962	M = 0.2548	M = 0.2497	M = 0.1995
	SD = 0.6045	SD = 0.5318	SD = 0.3056	SD = 0.1618	SD = 0.1346	SD = 0.0968
		T = 6	T = 0	T = 0	T = 0	T = 0
		Z = -0.943	Z = -2.201	Z = -2.201	Z = -2.201	Z = -2.201
		p = 0.345	p = **0.028**	p = **0.028**	p = **0.028**	p = **0.028**
E2L	M = 1.4142	M = 1.0750	M = 0.4872	M = 0.3118	M = 0.2022	M = 0.2052
	SD = 0.4605	SD = 0.4279	SD = 0.3551	SD = 0.1639	SD = 0.1163	SD = 0.0757
		T = 5	T = 0	T = 0	T = 0	T = 0
		Z = -1.153	Z = -2.201	Z = -2.201	Z = -2.201	Z = -2.201
		p = 0.249	p = **0.028**	p = **0.028**	p = **0.028**	p = **0.028**
E3L	M = 1.3507	M = 1.0330	M = 0.3735	M = 0.2482	M = 0.1750	M = 0.1627
	SD = 0.3906	SD = 0.4059	SD = 0.2142	SD = 0.0786	SD = 0.0669	SD = 0.0424
		T = 7	T = 0	T = 0	T = 0	T = 0
		Z = -0.734	Z = -2.201	Z = -2.201	Z = -2.201	Z = -2.201
		p = 0.463	p = **0.028**	p = **0.028**	p = **0.028**	p = **0.028**
E4L	M = 1.3803	M = 0.5148	M = 0.2900	M = 0.1783	M = 0.1533	M = 0.1250
	SD = 0.3016	SD = 0.1604	SD = 0.1407	SD = 0.0523	SD = 0.0635	SD = 0.0644
		T = 0	T = 0	T = 0	T = 0	T = 0
		Z = -2.201	Z = -2.201	Z = -2.201	Z = -2.201	Z = -2.201
		p = **0.028**	p = **0.028**	p = **0.028**	p = **0.028**	p = **0.028**
E5L	M = 1.1950	M = 0.4888	M = 0.2400	M = 0.2217	M = 0.0913	M = 0.0863
	SD = 0.4751	SD = 0.1760	SD = 0.1130	SD = 0.0578	SD = 0.0590	SD = 0.0733
		T = 0	T = 0	T = 0	T = 0	T = 0
		Z = -2.201	Z = -2.201	Z = -2.201	Z = -2.201	Z = -2.201
		p = **0.028**	p = **0.028**	p = **0.028**	p = **0.028**	p = **0.028**

BETA						
	Pre-MCAo	0h	4h	8h	12h	16h
E1L	M = 0.4318	M = 0.3548	M = 0.0813	M = 0.0432	M = 0.0422	M = 0.0402
	SD = 0.1554	SD = 0.1094	SD = 0.0366	SD = 0.0251	SD = 0.0194	SD = 0.0262
		T = 7	T = 0	T = 0	T = 0	T = 0
		Z = -0.734	Z = -2.201	Z = -2.201	Z = -2.201	Z = -2.201
		p = 0.463	p = **0.028**	p = **0.028**	p = **0.028**	p = **0.028**
E2L	M = 0.4337	M = 0.3510	M = 0.0978	M = 0.0393	M = 0.0207	M = 0.0137
	SD = 0.1260	SD = 0.0912	SD = 0.0331	SD = 0.0090	SD = 0.0059	SD = 0.0048
		T = 4	T = 0	T = 0	T = 0	T = 0
		Z = -1.363	Z = -2.201	Z = -2.201	Z = -2.201	Z = -2.201
		p = 0.173	p = **0.028**	p = **0.028**	p = **0.028**	p = **0.028**
E3L	M = 0.3937	M = 0.1617	M = 0.1180	M = 0.0455	M = 0.0242	M = 0.0197
	SD = 0.1931	SD = 0.0635	SD = 0.0517	SD = 0.0205	SD = 0.0149	SD = 0.0054
		T = 0	T = 0	T = 0	T = 0	T = 0
		Z = -2.201	Z = -2.201	Z = -2.201	Z = -2.201	Z = -2.201
		p = **0.028**	p = **0.028**	p = **0.028**	p = **0.028**	p = **0.028**
E4L	M = 0.4253	M = 0.2697	M = 0.1030	M = 0.0378	M = 0.0283	M = 0.0135
	SD = 0.1369	SD = 0.0724	SD = 0.0517	SD = 0.0079	SD = 0.0110	SD = 0.0059
		T = 0	T = 0	T = 0	T = 0	T = 0
		Z = -2.201	Z = -2.201	Z = -2.201	Z = -2.201	Z = -2.201
		p = **0.028**	p = **0.028**	p = **0.028**	p = **0.028**	p = **0.028**
E5L	M = 0.3783	M = 0.1325	M = 0.0593	M = 0.0267	M = 0.0185	M = 0.0123
	SD = 0.1332	SD = 0.0570	SD = 0.0250	SD = 0.0082	SD = 0.0042	SD = 0.0050
		T = 0	T = 0	T = 0	T = 0	T = 0
		Z = -2.201	Z = -2.201	Z = -2.201	Z = -2.201	Z = -2.201
		p = **0.028**	p = **0.028**	p = **0.028**	p = **0.028**	p = **0.028**

GAMMA						
	Pre-MCAo	0h	4h	8h	12h	16h
E1L	M = 0.0447	M = 0.0343	M = 0.0187	M = 0.0083	M = 0.0058	M = 0.0013
	SD = 0.0130	SD = 0.0171	SD = 0.0077	SD = 0.0021	SD = 0.0017	SD = 0.0006
		T = 4.5	T = 1	T = 0	T = 0	T = 0
		Z = -1.261	Z = -1.992	Z = -2.207	Z = -2.201	Z = -2.201
		p = 0.207	p = **0.046**	p = **0.027**	p = **0.028**	p = **0.028**
E2L	M = 0.0420	M = 0.0347	M = 0.0160	M = 0.0088	M = 0.0063	M = 0.0030
	SD = 0.0139	SD = 0.0119	SD = 0.0097	SD = 0.0023	SD = 0.0022	SD = 0.0021
		T = 6	T = 0	T = 0	T = 0	T = 0
		Z = -0.943	Z = -2.201	Z = -2.207	Z = -2.201	Z = -2.201
		p = 0.345	p = **0.028**	p = **0.027**	p = **0.028**	p = **0.028**
E3L	M = 0.0437	M = 0.0248	M = 0.0137	M = 0.0083	M = 0.0047	M = 0.0020
	SD = 0.0160	SD = 0.0075	SD = 0.0062	SD = 0.0012	SD = 0.0019	SD = 0.0009
		T = 0	T = 0	T = 0	T = 0	T = 0
		Z = -2.201	Z = -2.201	Z = -2.201	Z = -2.201	Z = -2.201
		p = **0.028**	p = **0.028**	p = **0.028**	p = **0.028**	p = **0.028**
E4L	M = 0.0462	M = 0.0233	M = 0.0162	M = 0.0103	M = 0.0055	M = 0.0022
	SD = 0.0126	SD = 0.0099	SD = 0.0082	SD = 0.0042	SD = 0.0024	SD = 0.0012
		T = 0	T = 0	T = 0	T = 0	T = 0
		Z = -2.201	Z = -2.201	Z = -2.201	Z = -2.201	Z = -2.201
		p = **0.028**	p = **0.028**	p = **0.028**	p = **0.028**	p = **0.028**
E5L	M = 0.0433	M = 0.0200	M = 0.0147	M = 0.0107	M = 0.0068	M = 0.0025
	SD = 0.0160	SD = 0.0073	SD = 0.0061	SD = 0.0029	SD = 0.0019	SD = 0.0010
		T = 1	T = 0	T = 0	T = 0	T = 0
		Z = -1.992	Z = -2.201	Z = -2.201	Z = -2.201	Z = -2.201
		p = **0.046**	p = **0.028**	p = **0.028**	p = **0.028**	p = **0.028**

Supplementary Table 10. Statistical analysis report - Changes in the frequency bands in the left electrodes after MCAo in the hypothermia group: Insulted hypothermic hemisphere. The Wilcoxon matched-pairs signed-rank test was applied to observe how the power spectra of the frequency bands changed 5 min after MCAo (0 h), at 4, 8, and 12 h, using the frequency bands collected 5 min prior to the MCAo as a baseline from the left injured hemisphere of normothermic brains. The statistically significant drop in the power spectra of the frequency bands was at ≤ 0.05. M, mean; SD, Standard deviation; T, t-test statistic; z, z score.
